# Site‐Selective Ligand Selection by Mutational Profiling for Covalent RNA Targeting

**DOI:** 10.1002/anie.202517243

**Published:** 2025-12-23

**Authors:** Phillip Yesley, Georgia Poulladofonou, Danny Incarnato, Willem A. Velema

**Affiliations:** ^1^ Institute for Molecules and Materials Radboud University Heyendaalseweg 135 Nijmegen 6525 AJ Netherlands; ^2^ Groningen Biomolecular Sciences and Biotechnology Institute University of Groningen Nijenborgh 7 Groningen 9747 AG Netherlands

**Keywords:** Covalent modification, Nucleic‐acid chemistry, Riboswitch, RNA probing, RNA targeting

## Abstract

To facilitate the design of RNA‐targeted covalent probes we report a bottom‐up, structure‐informed approach using mutational profiling. We designed a compact library of covalent probes based on the scaffold of Ribocil, a selective inhibitor targeting the bacterial FMN riboswitch and screened them against the FMN riboswitch aptamer from *Fusobacterium nucleatum*, *Bacillus subtilis*, *Escherichia coli*, and *Staphylococcus aureus*. This yielded Covacil, a highly base‐selective probe that covalently modifies the FMN riboswitch aptamer at low micromolar concentrations, within 10 minutes. We validate the site‐selectivity and covalency of the probe by competitive photo‐affinity labelling and mass‐spectrometry. When compared to non‐targeted covalent probes, Covacil displayed >1,000‐fold increased selectivity toward a specific base within the FMN riboswitch aptamer. Finally, we apply Covacil to total RNA and demonstrate that it maintains its base‐selective reactivity for the FMN riboswitch within the entire transcriptome.

## Graphical abstract

Using a mutational profiling approach an RNA‐targeted covalent probe was developed that is highly selective for the FMN riboswitch. The covalency was further supported by mass spectrometry experiments. The covalent probe selectively modifies the riboswitch at low micromolar concentrations within 10 min and remains functional when applied to an entire transcriptome.

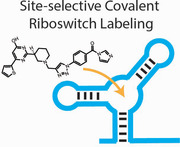

Catalyzed by the advent of next‐generation sequencing technologies, there is an expanding interest in the biology of non‐coding RNAs (ncRNAs).^[^ ^1^, ^2^, ^3^ ^]^ Through an enormous scientific effort, we now know that ncRNAs mediate a wide variety of biological functions, often enabled by specific RNA tertiary structures. Furthermore, structured RNAs can interact and selectively bind small molecules as exemplified by riboswitches^[^ ^4^, ^5^, ^6^, ^7^ ^]^ within the 5′UTR of mRNAs, whose interactions with metabolites and ions affect conformational changes that regulate the expression of their downstream genes. Beside riboswitches, small molecule interactions with RNA have become an expanding frontier in medicinal chemistry, as several structured RNAs are being pursued as novel drug targets, some of which for diseases so far thought to be undruggable.^[^ ^8^, ^9^, ^10^, ^11^, ^12^ ^]^ Diametrically, unintended interactions between known drugs and RNAs are also being discovered and could provide new perspectives in their unwanted side‐effects.^[^ ^13^ ^]^ Current tools for interrogating RNA‐small molecule interactions rely mainly on non‐covalent interactions between RNA and its ligands.^[^ ^14^ ^]^ Recently, strategies exploiting covalent bond formation between small molecule ligands and RNA are emerging.^[^ ^15^ ^]^

These approaches are of interest to afford bespoke research tools to decipher the biological function of RNA and discover druggable sites within the transcriptome. Additionally, they hold promise as covalent RNA targeting drugs.^[^ ^14^, ^16^ ^]^ This prospect is particularly exciting due to the noted successes of protein‐targeted covalent drugs.^[^ ^17^ ^]^ Several studies have reported on the use of photoreactive groups to form covalent adducts between small‐molecule probes and their RNA targets.^[^ ^18^, ^19^, ^20^, ^21^, ^22^ ^]^ These have successfully been used to interrogate interactions between RNA and its ligands in targeted as well as transcriptome‐wide experiments.^[^ ^19^, ^23^, ^24^ ^]^ While powerful, potential downsides are the requirement for UV activation that occurs with limited efficiency and prohibits further development into covalent inhibitors, and the oftentimes lack of site selectivity due to the high reactivity of the activated intermediates. More recently, RNA ligands bearing electrophilic warheads are being investigated that apart from their use as research tools have the potential as covalent RNA inhibitors (Figure 1a). Micura and coworkers elegantly demonstrated that placing an alkyl‐halide within the correct range to a nucleophilic N7 atom of guanine results in successful covalent bond formation, which they analyzed by mass spectrometry (MS).^[^ ^14^ ^]^ Similarly, Franceschi et al. developed a biologically active PreQ1 targeted probe using an icSHAPE based approach.^[^ ^25^ ^]^ Disney and colleagues reported on an attractive approach employing matrix‐assisted laser desorption/ionization‐time of flight (MALDI‐TOF) to screen for covalent RNA inhibitors.^[^ ^26^ ^]^ They also successfully targeted the FMN riboswitch using a glyoxal‐type warhead.^[^ ^27^ ^]^

**Figure 1:**
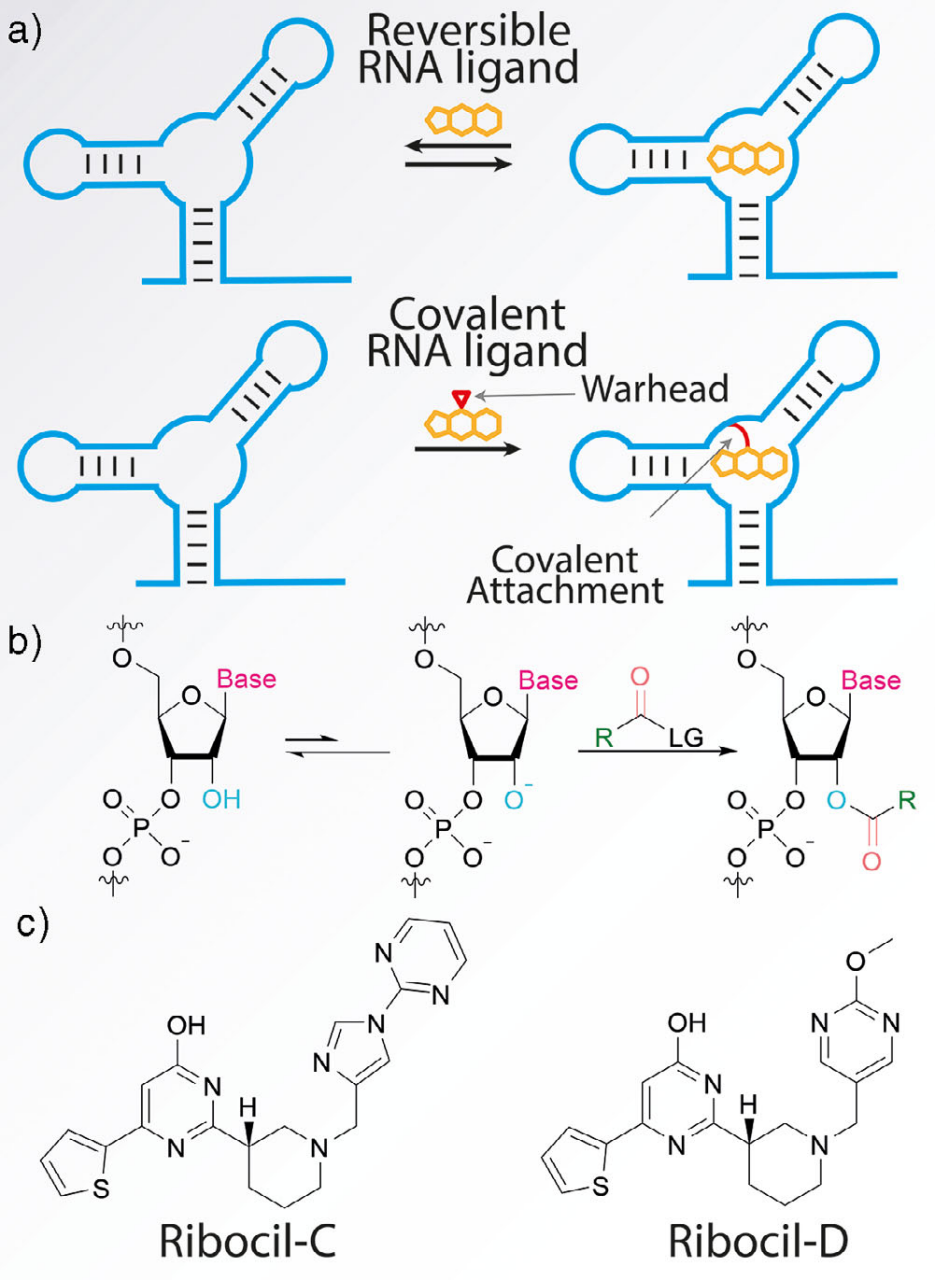
Schematic overview of covalent RNA targeting using 2′‐OH acylation and molecular structures of Ribocil derivatives. a) Comparison of reversible and covalent RNA‐binding small molecules. b) Concept of 2′‐OH modification. c) Structures of Ribocil‐based parent scaffolds for library of probes.

However, the successful design of covalent electrophilic RNA ligands remains challenging. The addition of the covalent group should not disrupt binding of the ligand, while the covalent group must still be sufficiently close to a reactive site on the RNA. An ideal screening platform informs on covalent‐bond formation as well as the exact site of modification. Although RNA 2′‐OH acylation has been extensively used in the past years, site‐selective RNA modification through rational design has remained challenging.

To complement the growing interest in RNA probes, this work demonstrates a generalizable pipeline to design covalent probes based on acyl‐imidazole warheads using mutational profiling.^[^ ^28^, ^29^ ^]^

As a starting point for the design of a compact library of covalent RNA‐targeted ligands we scrutinized the crystal structure of Ribocil‐D bound to the aptamer of the FMN riboswitch from *F. nucleatum*.^[^ ^30^ ^]^ To determine potential installation sites for an electrophilic warhead, priority was given to locations in close proximity to 2′‐OH groups that could be covalently modified (Figure 1b) and would not disrupt essential interactions between the ligand and aptamer. In this regard, the northern nitrogen‐containing heterocycle (Figure 1c) stood out for its proximity to several 2′‐OH groups and solvent exposure that could potentially accommodate an electrophilic warhead without disruption of ligand binding.^[^ ^31^ ^]^ Two sets of compounds were prepared based on the scaffolds of Ribocil‐C and Ribocil‐D (Figure 1c). In each case, the ortho, meta and para positions were tried as warhead positions. As electrophile, we elected to use *N*‐acyl imidazoles that have been shown to react well with 2′‐OH groups, yielding compounds (‐)**1**‐(‐)**6** (Figure 2a).^[^ ^32^, ^33^, ^34^, ^35^, ^36^ ^]^

**Figure 2:**
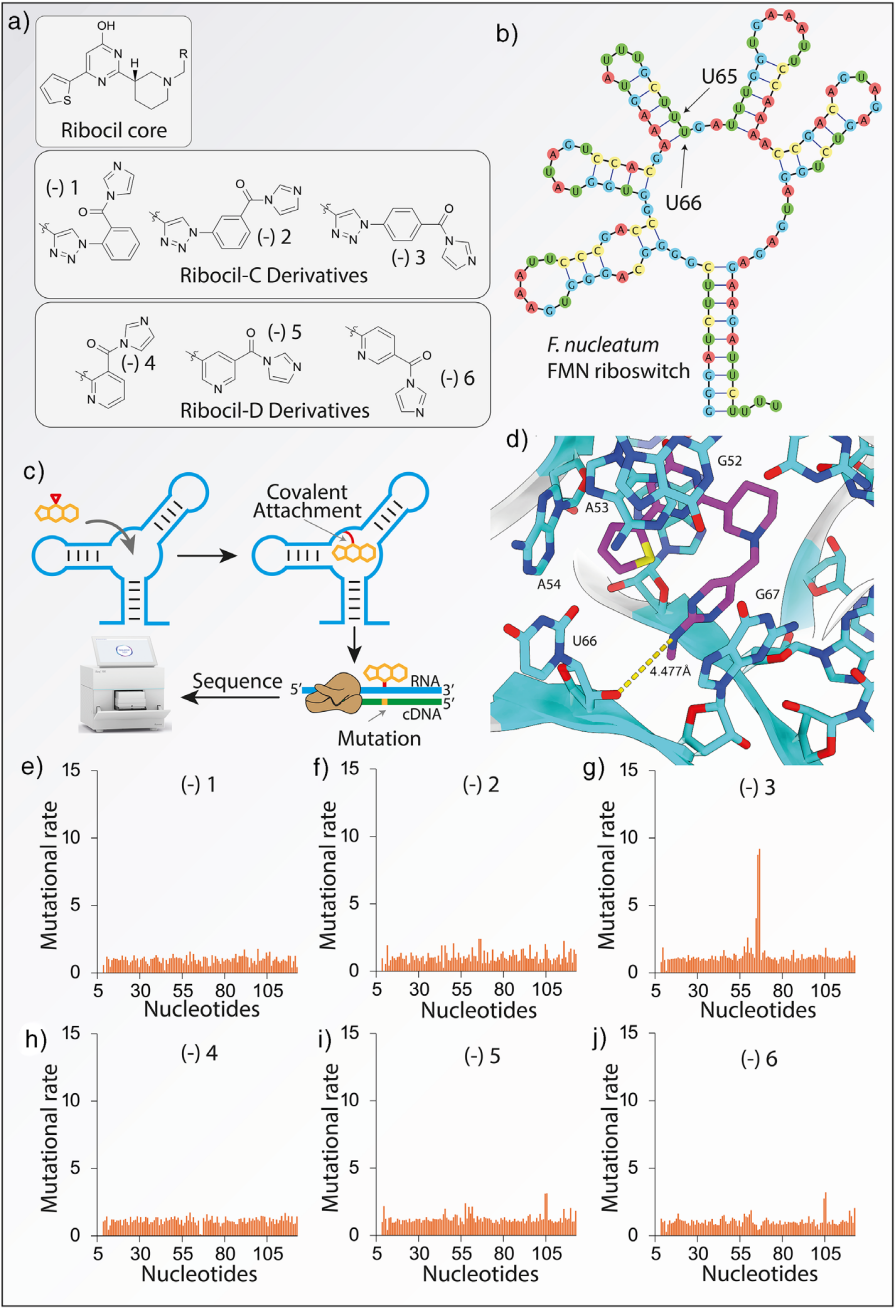
Screening of covalent probes using mutational profiling. a) Molecular structures of covalent Ribocil probes. b) Reported secondary structure of the *F. nucleatum* riboswitch aptamer. c) Overview of the mutational profiling pipeline to identify covalent probes. d). X‐ray crystallography structure of the *F. nucleatum* riboswitch aptamer bound to Ribocil‐D showing the proximity of the nitrogen‐containing heterocycle to bases U65 and U66 (PDB: 5KX9). e–j). Mutational rates by position for compound (‐)**1**‐ (‐)**6** (n = 1). Enhanced mutational rate is observed for (‐)**3** (g).

To evaluate our library, we first probed the FMN aptamer from *F. nucleatum* (Figure 2b). Compounds **1**–**6** were initially incubated at a relatively high concentration of 500 µM with in vitro folded RNA for 10 min at 37 °C after which the reaction was stopped (Figure 2c). RNA was folded according to published protocols that mimic physiological conditions (see Supporting Information).^[^ ^19^ ^]^ RNA was subjected to error‐prone reverse transcription with SuperScript‐II, a reverse transcriptase known to install mutations when it encounters modified 2′‐OH sites, in the presence of Mn^2+^ ions,^[^ ^37^ ^]^ allowing for mutations to be read by sequencing as opposed to drop‐off signals.

After aligning the sequencing reads to the FMN aptamer, each nucleotide's mutational rate was normalized to an untreated sample and analyzed to identify potential covalent modifications (Figure 2c). Notably, RNA treated with compound (‐)**3** displayed enhanced mutational rates at nucleotides U66 and adjacent U65. Based on the X‐ray structure^[^ ^38^ ^]^ (Figure 2d) the electrophilic carbonyl should be positioned immediately next to the 2′‐OH of U66, potentially explaining the observed result. Additionally (‐)**5** and (‐)**6** also showed modestly enhanced reactivities at positions A100 and U101. Due to their proximity to the binding site these may also be selective modifications.

None of the other scaffolds showed modifications, underlining the importance of positioning of the electrophilic warhead. Moreover, it suggests that the observed covalent modification likely stems from a selective interaction, rather than from unselective labeling expected from conventional SHAPE reagents.^[^ ^39^ ^]^

The FMN riboswitch aptamer is widespread among different bacteria.^[^ ^23^, ^24^ ^]^ To evaluate the covalent interaction between ligands **1**–**6** and other FMN riboswitch aptamers, we repeated the screen against the aptamer regions from *B. subtilis*, *E. coli* and *S. aureus*. Interestingly, an enhanced mutational rate was found for compound **3** at nucleotide U91 from the *B. subtilis* riboswitch aptamer (Figure S1). This nucleotide is reportedly related to U66 in the *F. nucleatum* riboswitch aptamer by sequence conservation^[^ ^40^ ^]^ that also displayed robust labeling with compound **3** (Figure 2g). To our knowledge, this is the first reported interaction between this aptamer and Ribocil. The FMN aptamers from *E. coli* and *S. aureus* showed no enhanced mutational rates after incubation with compounds **1–6** (Figures S2 and S3). Surprisingly, these aptamers are known to interact with Ribocil.^[^ ^38^, ^41^ ^]^

We propose that the lack of reactivity can potentially be explained by subtle differences in binding modes and the lack of nearby 2′‐OH groups. We further screened compound **3** against a panel of bacterial and human RNAs and DNA and observed no enhanced mutational rate (Figures S4–S8). Overall, the results underline the importance of scaffold design and ideal positioning of electrophiles to promote reactivity, which is in line with recent observations by Micura and coworkers.^[^ ^14^ ^]^

To further validate the covalent interaction between compound **3**, dubbed Covacil, and the FMN aptamer from *F. nucleatum* we performed mass spectrometry. The RNA aptamer was split in half as reported previously^[^ ^42^ ^]^ and refolded in 50 mM HEPES buffer, 10 mM MgCl_2_, pH 7.5 at 37 °C. 100 µM Covacil was added and incubated for 30 min at 37 °C and immediately analyzed by ESI‐MS. The treated sample showed an increased mass corresponding to Covacil added to the aptamer half containing U66 (Figure 3a,b), further supporting the covalent interaction between Covacil and the FMN riboswitch. Quantification of the mass spectra suggested an estimated conversion of 27% (Figure S9).

**Figure 3:**
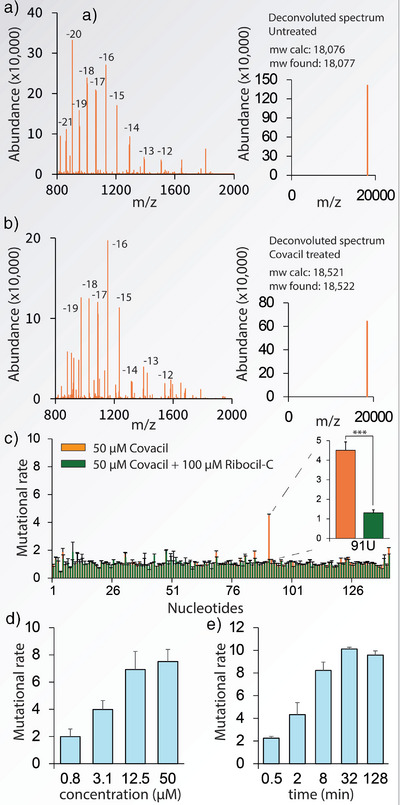
Mass spectrometry of covalent adduct and assessment of selective reactivity. a) Mass spectrum of untreated RNA. b) Mass spectrum of RNA treated with 100 µM Covacil, showing an increased mass corresponding to one covalent Covacil adduct. c) Mutational rate observed in the presence and absence of 100 µM Ribocil‐C (mean ± SD, n = 3). Statistical significance was calculated using an unpaired two‐tailed Student's *t* test (***p < 0.001). d) Observed mutational rate at increasing concentration of Covacil (mean ± SD, n = 3). e) Mutational rate observed at increasing incubation times (mean ± SD, n = 3).

To explore the power of mutational profiling to investigate covalent interactions, we devised a series of experiments to study the selectivity and reactivity of Covacil, toward the aptamer of the FMN aptamer from *B. subtilis*. To prove that the covalent interaction is mediated by the Ribocil ligand interacting with the aptamer we conducted a competition experiment with the unmodified parent compound Ribocil‐C. To do this, the aptamer was pre‐incubated with 100 µM Ribocil‐C for 20 min before incubation with 50 µM of Covacil for 10 min. The enhanced mutational rate observed at nucleotide U91 significantly decreased from ∼ 4.5 to 1 (background level), implying that the covalent interaction is indeed mediated through the Ribocil core of Covacil (Figure 3c). Moreover, to show that the RNA modification is contingent on correct folding of the aptamer, we incubated 50 µM Covacil for 10 min with unfolded RNA. The increased mutational rate at nucleotide U91 entirely disappeared, supporting the importance of correct aptamer folding to effectuate a covalent interaction (Figure S10).

To evaluate the sensitivity of mutational profiling and the efficiency of Covacil we incubated concentrations ranging from 0.8 µM to 50 µM with the FMN aptamer from *B. subtilis*. A concentration‐dependent effect was observed, with decreased mutational rate at lowering concentrations (Figure 3d). Aside from a clear decrease in signal in response to lower concentrations, we also observed clear modification at high nano‐molar concentrations.

To assess the speed of modification of Covacil we measured the modification of 100 µM at different time points. After only 8 min, close to maximal mutational rate was observed (Figure 3e), which is considerably shorter than the half‐life of Covacil, which was determined to be 65 min (Figure S11).

The enhancement of selectivity toward modification of the FMN aptamer provided by the Ribocil ligand was quantified by comparing mutational rates observed with Covacil and the commonly‐used SHAPE reagent NAI. At moderate concentrations of 100 µM, Covacil effectuates a robust mutational rate of 13.4 at the modified nucleotide, with no increased rate at other nucleotides (Figure 4a). A high concentration of 100 mM NAI is required to achieve a mutational rate of 6.6, implying an observed selectivity enhancement of > 1000‐fold for Covacil over NAI. Additionally, at this high concentration, NAI acylates many 2′‐OH groups within the aptamer (Figure 4b), while Covacil modifications appear targeted toward the 2′‐OH group of U91, further supporting the selectivity of Covacil. When NAI is used at the same concentration as Covacil (100 µM), no increased mutational rate is observed (Figures S12 and S13).

**Figure 4:**
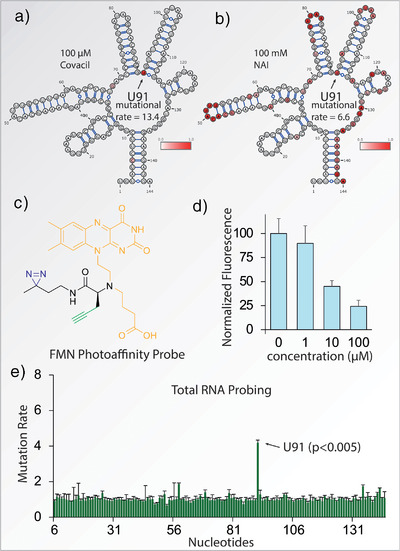
Selectivity enhancement and Total RNA probing. a) Reactivity profile of the *B. subtilis* aptamer treated with 100 µM Covacil. b) Reactivity profile of the *B. subtilis* aptamer treated with 100 mM NAI. c) Molecular structure of the FMN photoaffinity probe. d) Competitive binding at increasing concentrations of Covacil quantified by gel electrophoresis (mean ± SD, n = 3) (Uncropped gels in Figure S7). e) Mutational rate observed when total RNA isolated from *B. subtilis* is treated with 500 µM Covacil (mean ± SD, n = 3).

We further determined whether Covacil can competitively bind to the FMN aptamer using a non‐sequencing‐based method. Employing a previously‐described photoaffinity labeling probe based on the FMN scaffold^[^ ^18^ ^]^ (Figure 4c) we quantified competitive binding using gel electrophoresis. At low micromolar concentrations, Covacil displayed robust competitive binding, comparable to the Ribocil‐C parent compound (Figure 4d and Figure S14). To study a potential enhancement in binding affinity due to the covalent nature of Covacil, its competitive binding was compared to a non‐covalent ester analogue (Figure S15). At 1‐100 µM Covacil appeared more efficient in competing with the photoaffinity probe, hinting at a possible affinity enhancement due to the covalent interaction. Since Ribocil itself displays no antibacterial activity against *B. subtilis*, we found Covacil to be inactive as well (Figure S16)

Lastly, we studied the performance of Covacil within an entire bacterial transcriptome. Total RNA was isolated from *B. subtilis* and refolded in 25 mM HEPES, pH 7.5, 20 mM KCl, 5 mM MgCl_2_ and treated with Covacil for 10 min. Using primers targeting the FMN riboswitch, we amplified the aptamer region out of the transcriptome and subjected it to the mutational profiling pipeline. Remarkably, an increased mutational rate was still present at nucleotide U91, emphasizing the ability of Covacil to covalently interact with the FMN aptamer within a complex mixture of RNAs present in the transcriptome (Figure 4e).

Overall, we believe that our results suggest that a structure‐first approach, via in vitro mutational profiling is an effective strategy to designing covalent RNA‐targeted probes. Furthermore, mutational profiling can be used to describe the kinetics of these covalent interactions, whilst not being limited by the size of the RNA. We think that such experiments could prove highly informative in the design of therapeutic RNA‐targeted covalent inhibitors. Indeed, given very recent advances in RNA‐targeted chemistries and their apparent compatibility with mutational profiling, the stage is set for exciting development.^[^ ^26^, ^43^ ^]^

## Supporting Information

The authors have cited additional references within the Supporting Information.^[^ ^44^, ^45^, ^46^, ^47^ ^]^

## Conflict of Interests

The authors declare no conflict of interest.

## Supporting information



Supporting Information

## Data Availability

The data that support the findings of this study are available from the corresponding author upon reasonable request.
