# Supplementary material for: Site‐Selective Ligand Selection by Mutational Profiling for Covalent RNA Targeting
Source: Angew Chem Int Ed Engl. 2025 Dec 23;65(6):e17243. doi: 10.1002/anie.202517243 (PMC12865241; doi:10.1002/anie.202517243)
Supplement: Supplementary file 1 — Supporting Information [file ANIE-65-e17243-s001.docx]

**Supporting Information**

Site-selective Ligand Selection by Mutational Profiling for Covalent RNA Targeting

Phillip Yesley,^[a]^ Georgia Poulladofonou,^[a]^ Danny Incarnato^[b]^ and Willem A. Velema*^[a]^

[a]Institute for Molecules and Materials
Radboud University
Heyendaalseweg 135, 6525 AJ Nijmegen
E-mail: willem.velema@ru.nl

[b]Groningen Biomolecular Sciences and Biotechnology Institute
University of Groningen
Nijenborgh 7, 9747 AG Groningen

**Table of contents**

Supporting Figures (S1-S16)………………………………………………………………………...P2 Methods And Materials…………………………………………………………………………...…P18 DNA Template Preparation……………………………………………………………………….....P18 In-Vitro RNA Probing and Mutational Profiling…………………………………………………….P18 Mass Spectrometry…………………………………………………………………………………..P19 In-Vitro Transcriptome-Wide Probing………………………………………………………………P19 Sequencing Analysis………………………………………………………………………………....P19 Competitive Labelling……………………………………………………………………………….P20

Bacterial Growth Curves…………………………………………………………………………….P20

Table 1: Template Sequences………………………………………………………………………..P20 Table 2: Riboswitch Constructs……………………………………………………………………...P22 Table 3: Primers……………………………………………………………………………………...P23 Table 4: Riboswitch RNA…………………………………………………………………………...P25 General Synthesis……………………………………………………………………………………P26 Spectra……………………………………………………………………………………………….P39 References…………………………………………………………………………..………...……..P64

**Figure S1**. a) Secondary structure of the FMN riboswitch from *Bacillus subtilis* (*B. subtilis*). b) Normalized reactivities of the FMN riboswitch from *B. subtilis* probed with compounds **1-6** at 500 µM (n = 1). Compound 3 shows an elevated reactivity rate at position U106 (U91 in the secondary structure and main text)

**Figure S2**. a) Secondary structure of the FMN riboswitch from *Escherichia coli* (*E. coli*). b) Normalized reactivities of the FMN riboswitch from *E. coli* probed with compounds **1-6** at 500 µM (n = 1).

**Figure S3**. a) Secondary structure of the FMN riboswitch from *Staphylococcus aureus* (*S. aureus*). b) Normalized reactivities of the FMN riboswitch from *S. aureus* probed with compounds **1-6** at 500 µM (n = 1).

**Figure S4**. a) Secondary structure of the ThiC riboswitch from *Escherichia coli* (*E. Coli*). b) Normalized reactivities of the ThiC riboswitch probed by 500 µM Covacil, showing no increased reactivity at any position (n = 1).

**Figure S5**. a) Secondary structure of the Smk riboswitch from *Enterococcus faecalis* (*E. faecalis*). b) Normalized reactivities of the Smk riboswitch probed by 500 µM Covacil, showing no increased reactivity at any position (n = 1).

**Figure S6**. a) Secondary structure of the Guanine riboswitch from *Bacillus subtilis* (*B. subtilis*). b) Normalized reactivities of the Guanine riboswitch probed by 500 µM Covacil, showing no increased reactivity at any position (n = 1).

**Figure S7**. a) Predicted Secondary structure of the 4270-4425 segment of the human 28S ribosome. b) Normalized reactivities of the amplified region from refolded total RNA from HEK293 cells, probed by 500 µM Covacil, showing no increased reactivity at any position. (n = 1)

**Figure S8**. Covacil does not covalently interact with DNA. a) Sequence and molecular weight of DNA used. b) Chromatogram of Covacil in Folding Buffer (15 mM MgCl2, 100mM KCl and 50 mM HEPES, pH 8.0) add buffer components).c) Chromatogram of DNA 1, treated with 100 µM Covacil. No new peaks in the chromatogram or mass spectrum are observed, implying that Covacil does not covalently label DNA 1. d) Mass spectrum of eluted peak 3 showing only mass peaks corresponding to unmodified DNA 1

**Figure S9**. Estimation of covalent labelling yield with Covacil. a) Secondary structure of the aptamer of the FMN riboswitch from *F. Nucleatum*. The aptamer is made of two halves, S1 (red) and S2 (blue) as reported earlier^1^ b) Chromatogram of the FMN aptamer displayed in panel a treated with 100 µM Covacil. See section ‘Mass Spectrometry’ for exact conditions. c) Mass spectrum of eluted peak 1 showing both S1 and S2. b) Mass spectrum of eluted peak 2 showing peaks corresponding to the mass of S2 + Covacil.

Peak 1 contains both unmodified S1 and S2 that elute together. Peak 2 elutes later and contains S2 modified with Covacil only. The total area = 43985.17 + 6775.48 = 50760.65 Approximately half the area (=25380.32) corresponds to S2, of which 6775.48 is covalently modified. Following this we estimate the conversion to be ~ 6775.48/25380.32 x 100 = 27%. This aligns with a very recently reported covalent RNA ligand that displayed ~33% modification^2^.

**Figure S10**. Mutational rate of folded/unfolded of the FMN riboswitch from *B. subtilis.* RNA was re-folded in presence of 15 mM MgCl_2_ (folded) or without (unfolded), probed with 50 µM Covacil (mean, n = 3 ).

**Figure S11**. a) Reaction scheme of the hydrolysis reaction of racemic Covacil. b) Normalized area of proton (H-NMR taken in 1:1 D_2_O, D_6_-DMSO, highlighted in red in panel a) which diminishes due to a shift in response to hydrolysis. c) A pseudo-first order fit gives an estimate half-life in aqueous solution of 65 minutes.

**Figure S12**. Reactivity plot of the FMN riboswitch from *B. subtilis* treated with 100 µM NAI.

**Figure S13.** Normalized reactivity of the RibD FMN aptamer treated with a) 100mM of NAI (n = 1) and b) 100µM Covacil (n = 1).

**Figure S14**. Labelling experiment of the FMN riboswitch from *B. subtilis* with an FMN-based diazirine probe (See Methods Section). Top (a) are the uncropped FAM fluorescent intensities (Lanes 1,2,3: Competition with 100 µM Ribocil-C, Lane: 4,5,6: Competition with Covacil 100 µM, Lanes: 7,8,9 Competition with Covacil 10 µM, , Lanes: 10,11,12: Competition with Covacil 1 µM, Lanes 13,14,15: No competition). Bottom (b) are the respective uncropped SYBR gold stains.

**Figure S15.** Covacil shows stronger competition with FMN photoaffinity label than the non-covalent methyl ester at three different concentrations. Graph in a) shows the fluorescent labelling, normalized to DMSO, in presence of 100,10 and 1 µM Covacil (f) (red) or Methyl ester (g) (blue) (mean ± SD, n = 3). Cropped Scans in (b-e) of the RNA on gel. Lanes 1, 2, 3 is treated with 1 µM, lanes 4, 5, 6 with 10 µM, lanes 7, 8, 9 with 100 µM and lanes 10, 11, 12 with DMSO. Rows b and d are RNA competed with Covacil or Methylate respectively, below (c and e) are the corresponding SYBR gold stains. Uncropped scans (h-k) correspond to Covacil labelling (h), Covacil SYBR gold stain (i), Covacil methyl ester (j) and Covacil methyl ester SYBR gold staining (k).

.

**Figure S16.** Covacil (a) and its methyl ester (Cov-Me) (b) do not have any significant antimicrobial effect on *Bacillus subtillis*, as seen by the growth curves at concentrations ranging from 150 nM to 200 µM (mean ± SD, n = 3)

**Methods and Materials**

**DNA Template Preparation**

All templates for the production of RNA aptamers were produced from in-house DNA sequences. The FMN riboswitch from *B. subtills* was used in previous work^3^. The FMN riboswitch from *E. coli,* was amplified from an IVTT plasmid first used by the Pedrolli lab^4^ (Table 1). The *S. aureus* aptamer was amplified from a gBlock^TM^ (ordered from IDT) containing the aptamer sequence (Table 1). The *F. nucleatum* template (Table 2) was ordered from IDT as a gBlock^TM^. PCR was carried out using standard PFU conditions. Briefly, to 20 µL of template DNA (10-100 ng): 1 µL of forward primer (100 µM), 1 µL reverse primer (100 µM) (see table 3), 10 µL X10 Phusion PCR buffer (200 mM Tris, 100 mM (NH4)2SO4, 100 mM KCl, 1 mg/ml BSA, 1% TRiton X-100, 20 mM MgSO_4_.7H20), 2 µL dNTPs (10 mM each), 2 µL X20 PFU polymerase (1 U/µL, produced in house) and 64 µL nuclease-free water. PCR reactions were carried out on a Biometra™ advanced thermocycler using typical Phusion conditions over 25 cycles; 60 s initial denaturation at 98 °C, 30 s cycle denaturation 98 °C, 20 s cycle annealing 63 °C, 30 s cycle extension 72 °C, final extension 5 min 72 °C. If necessary, DNA was isolated using the QIAQuick^®^ Gel Extraction kit (cat. 28704), as per manufacturer’s instructions. To improve yield, PCR was repeated on purified product, if necessary.

**RNA Production**

All RNA aptamers used for probing experiments were produced by T7 *in-vitro* transcription. Briefly, 250 ng of DNA template (Table 1) was resuspended in T7 transcription buffer ( 40 mM Tris-HCl, 25 mM MgCl2, 1 mM Spermidine, 5 mM DTT, 4 mM rNTPs, 5 mM GMP, pH 8.1), to a final volume of 225 µL. 25 µL of T7 RNA polymerase (250 U/µL, produced in-house) was added, and transcription proceeded at 37 °C, for 4 hours. DNA was digested by adding 5 µL of DNAse I (Zymo research™, E1010). RNA was extracted with Trizol^®^, as per manufacturer’s instructions, obtaining 300 µL aqueous RNA solution. RNA was further purified by isopropanol precipitation, and subsequently quantified by Qubit^TM^ RNA High Sensitivity assay (Thermofisher Q32852).

***In-Vitro* RNA Probing And Mutational Profiling**

RNA was re-folded as done previously^1^. Briefly, RNA was resuspended in 45 µL of nuclease-free water to a final concentration of 10-40 nM. RNA was denatured by heating to 95 °C for 2 min and immediately snapped cooled on ice. The solution was supplemented with 5 µL X10 RNA folding buffer (150 mM MgCl2, 1M KCl and 500mM HEPES, pH 8.0), and incubated at room temperature for 5 min. To probe re-folded the 4270-4425 region of the human 28S ribosome, 600 ng of Human total RNA obtained from HEK293 cells as described previously and refolded as described in the in-vitro transcriptome-wide probing section in 50 µL.

RNA was probed by adding freshly activated probe (see Synthesis section) to the RNA and incubating for 10 min, whilst shaking at 37°C. For experiments with activated 2-methylnicotinic acid (NAI), the acid was first activated at 1M, as done previously and then used in the same way.^5^ The mixture was quenched by adding 100 µL of RNA Binding buffer (Zymo RNA Clean-up 5^®^, R1013) followed by 150 µL ethanol and immediately passed through a spin column, by following manufacturers instructions, eluting in 9.5 µL of nuclease-free water. Reverse transcription primers containing Illumina^®^ sequencing adaptors (see table 3) were annealed by adding 1 µL primer (100 µM) and 1 µL dNTPs (10 mM), heating to 95 °C for 2 min and immediately snap cooled. To the annealed mixture, was added 9.5 µL of error prone Superscript II reaction mixture: 4 µL first strand buffer x5 (250 mM Tris, 375 KCl, pH 8), 2 µL DTT (100 mM), 1 µL Superscript II 200 U/µL (Invitrogen™, 18064014**),** 0.5 µL Water and 1 µL MnCl_2_ (120 mM). Reverse-transcription was carried out over 2 hours at 42°C.

Resultant cDNA libraries were amplified with primers containing Illumina^®^ sequencing adaptor overhangs. Reverse primers were the same as those used for reverse-transcription. Briefly, to 20 µL of cDNA was added: 1 µL of forward primer (100 µM) , 1 µL reverse primer (100 µM), 10 µL X10 Phusion PCR buffer (200 mM Tris, 100m M (NH4)_2_SO_4_, 100 mM KCl, 1 mg/mL BSA, 1% TRiton X-100, 20mM MgSO_4_.7H_2_0), 2 µL dNTPs (10 mM each), 2 µL x20 PFU polymerase (1 U/µL, produced in house) and 64 µL nuclease-free water. PCR reactions were carried out on a Biometra™ Tadvanced thermocycler using typical Phusion conditions over 25 cycles; 60s initial denaturation at 98 °C, 30 s cycle denaturation 98 °C, 20 s cycle annealing 63 °C, 30 s cycle extension 72 °C, final extension 5 min 72 °C. If necessary, DNA was isolated using the QIAQuick^®^ Gel Extraction kit (cat. 28704), as per manufacturer’s instructions. Purified DNA was quantified using the High Sensitivity dsDNA quibit assay (cat Q33231**).** 1 ng of DNA was then used as input for the NEBNext^®^ ultra II DNA Library Prep kit for Illumina^®^ (E7645S), using the NEBNext^®^ Multitplex oligos for Illumina (E7600S) as per manufacturer’s instructions. The indexed libraries were pooled, and sequenced on an Illumina Iseq100, following the manufacturer’s guidelines.

**Mass Spectrometry**

The split aptamer from *F. nucleatum* (Table 4) was refolded by dissolving both strands in 50 mM HEPES buffer, 10 MgCl_2_, pH 7.5 at a final concentration of 1 µM. The solution was heated to 95 °C for 5 min and slowly cooled to 37 °C. 100 µM Covacil (100x dilution of 10 mM DMSO stock) was added and incubated for 10 min, after which it was directly injected on an Agilent 1260 Infinity II LC-MSD equipped with AP-ESI Ion Source. Data processing and spectral deconvolution was performed with OpenLAB CDS2 software.

**In-Vitro Transcriptome-wide probing**

*B. subtilis* wild type was cultured overnight in LB medium supplemented with 5 mM MgSO_4_ and 20 mM Glucose. Cells were lysed by pelleting 2 mL of overnight culture and resuspending in 250 µL of TE buffer (10mM Tris Base, 0.1 mM EDTA pH 8) supplemented with 20 mg/mL Lysozyme and 0.5 µL SUPERase·In™ RNase Inhibitor (20 U/μL). For optimal lysis, cells were incubated on shaker for 20 min at 37 °C. RNA was extracted from lysate with 700 µL of Trizol^®^, as per manufacturers instructions. The aqueous phase, containing total RNA, was passed through an RNA clean-up column as per manufacturers instructions (Zymo RNA Clean-up 5^®^, R1013), eluting in 18 µL. Any contaminant DNA was digested by subjecting the eluted total RNA to TURBO^TM^ DNAse (Invitrogen catalogue no. AM2238) treatment as per manufacturers instructions. Reaction was subsequently cleaned by column, as done previously. Total RNA was quantified by Quibit^TM^ .RNA High Sensitivity assay. Quality of RNA was assessed by Agarose 2%.

To re-fold RNA *in-vitro*, 6 µg of total RNA was resuspended in 89 µL of nuclease free water. RNA was denatured by heating to 98°C for 2 min and immediately cooled on ice. To initiate secondary structure formation, 10 μl of ice-cold 10X Folding Buffer (250 mM HEPES pH 7.5; 2 M KCl ) were then added and samples were incubated at 37°C for 15 min. For tertiary structure formation 1 µL 500 mM MgCl_2_ (pre-warmed at 37 °C) was then added, and folding was allowed to proceed for another 15 min.

After folding, RNA was probed with 500 µM Covacil. Probed RNA was purified with a clean-up column as per manufacturers instructions (Zymo RNA Clean-up 5^®^, R1013) and quantified by Quibit^TM^. 250 ng of RNA were used as input for library preparation, exactly as done previously using appropriate primers.

**Sequencing Analysis**

All sequencing data was analyzed using the RNA framework^6^. Reads were aligned using *rf-map* command using the *“very sensitive local alignment”* bowtie2 preset. Reads with undetermined bases were discarded using -ctn 0, low quality bases (Phred < 20) were trimmed from the 5’ end with -cq5 20. After alignment, mutations were counted using the *rf-count* command, using the -m parameter. Deletions and insertions were ignored with -ni and -nd respectively. Mutations were only counted if surrounding bases had a Phred > 20, using -es with default quality parameters. Raw mutation counts and coverage were retrieved by running *rf-rctools* *view* on the previously produced count files. Mutational rates were scored in python, by calculating the fold change per base with respect to an untreated sample. For visualization normalized mutational rates were discretized in ranges of 2.5. High reactivities, above 10, were assigned 1. Discretized reactivities were overlayed on RNA structures using the VARNA toolkit.^7^

**Competitive Labelling with Photoaffinity Probe**

Site-specific interaction with the FMN riboswitch from *B. subtillis* was corroborated as done in our lab previously^3^. Briefly, the aptamer was resuspended to 1 µM in 60 µL of nuclease-free water. The RNA was subsequently denatured by heating to 95 ºC and immediately cooled on ice. The RNA was folded by adding 6 µL X10 Folding buffer (150 mM MgCL2, 1M KCl and 500mM HEPES, pH 8.0). The FMN PAL probe was added in 0.6 µL (20 %DMSO) to give a final concentration of 20 µM, the mixture was incubated at 37 ºC for 10 min. 0.6 µL of Covacil or Covacil methyl ester in DMSO was then added and the mixture was further incubated for 10 min. After the competition had taken place, the PAL probe was cross-linked to the RNA with 365 nm UV light in a UV Stratalinker for 15 min. The RNA was ethanol precipitated, by standard procedure^8^, and resuspended in 18 µL of Nuclease-free water. A ten-fold concentrated click mixture (2 µL) in water was added to the RNA to obtain final concentrations of 0.5 mM CuSO4, 5.0 mM sodium ascorbate, 2.5 mM Tris-(3- hydroxypropyltriazolylmethyl)amine and 0.475 mM fluorescein azide. The click reaction was allowed to proceed for 1 hour at 37 ºC. The labelled RNA was purified by ethanol precipitation and visualized on a 8% PAGE-denaturing gel using a Typhoon Amersham at 488 nm excitation.

**Bacterial Growth Curves**

To investigate the cytotoxicity of Covacil and its methyl ester towards *B. subtilis*, growth curves were measured. Briefly, *B. subtilis* was cultured in CSE minimal medium, overnight, under aerobic conditions, as done previously^9^. On a flat-bottomed transparent 96-well Greiner plate, freshly prepared Covacil or its methyl ester were prepared using a 2x fold dilution series in CSE minimal medium. Immediately afterwards, diluted cell-culture was added on top, to give an approximate OD600 of 0.025 in 100 µL. The plate was transferred into a Biotek 800 TS platereader, where absorbance was followed overnight, measuring every 10 minutes, at 37 °C.

**Table 1:**

**Template Sequences**

| **Sequence Name** | **Sequence (Aptamer in Bold)** |
| --- | --- |
| ***E. coli FMN Riboswitch  (psroGp2‑FMN‑luc)*** | CCTGACGTCTAAGAAACCATTATTATCATGACATTAACCTATAAAAATAGGCGTATCACGAGGCCCTTTCGTCTTCAAGAATTCTGGCGAATCCTCTGACCAGCCAGAAAACGACCTTTCTGTGGTGAAACCGGATGCTGCAATTCAGAGCGCCAGCAAGTGGGGGACAGCAGAAGACCTGACCGCCGCAGAGTGGATGTTTGACATGGTGAAGACTATCGCACCATCAGCCAGAAAACCGAATTTTGCTGGGTGGGCTAACGATATCCGCCTGATGCGTGAACGTGACGGACGTAACCACCGCGACATGTGTGTGCTGTTCCGCTGGGCATGCCAGGACAACTTCTGGTCCGGTAACGTGCTGAGCGGCGTTGCTGCCGCTAATCATTAGCGTATAGTGAATCCGCTTAAAGCTTGC**TTATTCTCAGGGCGGGGCGAAATTCCCCACCGGCGGTAAATCAACTCAGTTGAAAGCCCGCGAGCGCTTTGGGTGCGAACTCAAAGGACAGCAGATCCGGTGTAATTCCGGGGCCGACGGTTAGAGTCCGGATGGGAGAGAGTAAC**GATTCTGTCGGGCATGGACCCGCTCACGTTATTTTGGCTATATGCCGCCACTCCTAAGACTGCCCTGATTCTGGTAACCATAATTTTAGTGAGGTTTTTTTACCATGAATCAGACGCTACTTTGGATCCAAGCAGAGTTGGAAGACGCCAAAAACATAAAGAAAGGCCCGGCGCCATTCTATCCTCTAGAGGATGGAACCGCTGGAGAGCAACTGCATAAGGCTATGAAGAGATACGCCCTGGTTCCTGGAACAATTGCTTTTACAGATGCACATATCGAGGTGAACATCACGTACGCGGAATACTTCGAAATGTCCGTTCGGTTGGCAGAAGCTATGAAACGATATGGGCTGAATACAAATCACAGAATCGTCGTATGCAGTGAAAACTCTCTTCAATTCTTTATGCCGGTGTTGGGCGCGTTATTTATCGGAGTTGCAGTTGCGCCCGCGAACGACATTTATAATGAACGTGAATTGCTCAACAGTATGAACATTTCGCAGCCTACCGTAGTGTTTGTTTCCAAAAAGGGGTTGCAAAAAATTTTGAACGTGCAAAAAAAATTACCAATAATCCAGAAAATTATTATCATGGATTCTAAAACGGATTACCAGGGATTTCAGTCGATGTACACGTTCGTCACATCTCATCTACCTCCCGGTTTTAATGAATACGATTTTGTACCAGAGTCCTTTGATCGTGACAAAACAATTGCACTGATAATGAATTCCTCTGGATCTACTGGGTTACCTAAGGGTGTGGCCCTTCCGCATAGAACTGCCTGCGTCAGATTCTCGCATGCCAGAGATCCTATTTTTGGCAATCAAATCATTCCGGATACTGCGATTTTAAGTGTTGTTCCATTCCATCACGGTTTTGGAATGTTTACTACACTCGGATATTTGATATGTGGATTTCGAGTCGTCTTAATGTATAGATTTGAAGAAGAGCTGTTTTTACGATCCCTTCAGGATTACAAAATTCAAAGTGCGTTGCTAGTACCAACCCTATTTTCATTCTTCGCCAAAAGCACTCTGATTGACAAATACGATTTATCTAATTTACACGAAATTGCTTCTGGGGGCGCACCTCTTTCGAAAGAAGTCGGGGAAGCGGTTGCAAAACGCTTCCATCTTCCAGGGATACGACAAGGATATGGGCTCACTGAGACTACATCAGCTATTCTGATTACACCCGAGGGGGATGATAAACCGGGCGCGGTCGGTAAAGTTGTTCCATTTTTTGAAGCGAAGGTTGTGGATCTGGATACCGGGAAAACGCTGGGCGTTAATCAGAGAGGCGAATTATGTGTCAGAGGACCTATGATTATGTCCGGTTATGTAAACAATCCGGAAGCGACCAACGCCTTGATTGACAAGGATGGATGGCTACATTCTGGAGACATAGCTTACTGGGACGAAGACGAACACTTCTTCATAGTTGACCGCTTGAAGTCTTTAATTAAATACAAAGGATATCAGGTGGCCCCCGCTGAATTGGAATCGATATTGTTACAACACCCCAACATCTTCGACGCGGGCGTGGCAGGTCTTCCCGACGATGACGCCGGTGAACTTCCCGCCGCCGTTGTTGTTTTGGAGCACGGAAAGACGATGACGGAAAAAGAGATCGTGGATTACGTCGCCAGTCAAGTAACAACCGCGAAAAAGTTGCGCGGAGGAGTTGTGTTTGTGGACGAAGTACCGAAAGGTCTTACCGGAAAACTCGACGCAAGAAAAATCAGAGAGATCCTCATAAAGGCCAAGAAGGGCGGAAAGTCCAAATTGTAACTCGAGCAAAGCCCGCCGAAAGGCGGGCTTTTCTGTGTCGACCGATGCCCTTGAGAGCCTTCAACCCAGTCAGCTCCTTCCGGTGGGCGCGGGGCATGACTATCGTCGCCGCACTTATGACTGTCTTCTTTATCATGCAACTCGTAGGACAGGTGCCGGCAGCGCTCTTCCGCTTCCTCGCTCACTGACTCGCTGCGCTCGGTCGTTCGGCTGCGGCGAGCGGTATCAGCTCACTCAAAGGCGGTAATACGGTTATCCACAGAATCAGGGGATAACGCAGGAAAGAACATGTGAGCAAAAGGCCAGCAAAAGGCCAGGAACCGTAAAAAGGCCGCGTTGCTGGCGTTTTTCCATAGGCTCCGCCCCCCTGACGAGCATCACAAAAATCGACGCTCAAGTCAGAGGTGGCGAAACCCGACAGGACTATAAAGATACCAGGCGTTTCCCCCTGGAAGCTCCCTCGTGCGCTCTCCTGTTCCGACCCTGCCGCTTACCGGATACCTGTCCGCCTTTCTCCCTTCGGGAAGCGTGGCGCTTTCTCATAGCTCACGCTGTAGGTATCTCAGTTCGGTGTAGGTCGTTCGCTCCAAGCTGGGCTGTGTGCACGAACCCCCCGTTCAGCCCGACCGCTGCGCCTTATCCGGTAACTATCGTCTTGAGTCCAACCCGGTAAGACACGACTTATCGCCACTGGCAGCAGCCACTGGTAACAGGATTAGCAGAGCGAGGTATGTAGGCGGTGCTACAGAGTTCTTGAAGTGGTGGCCTAACTACGGCTACACTAGAAGAACAGTATTTGGTATCTGCGCTCTGCTGAAGCCAGTTACCTTCGGAAAAAGAGTTGGTAGCTCTTGATCCGGCAAACAAACCACCGCTGGTAGCGGTGGTTTTTTTGTTTGCAAGCAGCAGATTACGCGCAGAAAAAAAGGATCTCAAGAAGATCCTTTGATCTTTTCTACGGGGTCTGACGCTCAGTGGAACGAAAACTCACGTTAAGGGATTTTGGTCATGAGATTATCAAAAAGGATCTTCACCTAGATCCTTTTAAATTAAAAATGAAGTTTTAAATCAATCTAAAGTATATATGAGTAAACTTGGTCTGACAGTTACCAATGCTTAATCAGTGAGGCACCTATCTCAGCGATCTGTCTATTTCGTTCATCCATAGTTGCCTGACTCCCCGTCGTGTAGATAACTACGATACGGGAGGGCTTACCATCTGGCCCCAGTGCTGCAATGATACCGCGAGACCCACGCTCACCGGCTCCAGATTTATCAGCAATAAACCAGCCAGCCGGAAGGGCCGAGCGCAGAAGTGGTCCTGCAACTTTATCCGCCTCCATCCAGTCTATTAATTGTTGCCGGGAAGCTAGAGTAAGTAGTTCGCCAGTTAATAGTTTGCGCAACGTTGTTGCCATTGCTACAGGCATCGTGGTGTCACGCTCGTCGTTTGGTATGGCTTCATTCAGCTCCGGTTCCCAACGATCAAGGCGAGTTACATGATCCCCCATGTTGTGCAAAAAAGCGGTTAGCTCCTTCGGTCCTCCGATCGTTGTCAGAAGTAAGTTGGCCGCAGTGTTATCACTCATGGTTATGGCAGCACTGCATAATTCTCTTACTGTCATGCCATCCGTAAGATGCTTTTCTGTGACTGGTGAGTACTCAACCAAGTCATTCTGAGAATAGTGTATGCGGCGACCGAGTTGCTCTTGCCCGGCGTCAACACGGGATAATACCGCGCCACATAGCAGAACTTTAAAAGTGCTCATCATTGGAAAACGTTCTTCGGGGCGAAAACTCTCAAGGATCTTACCGCTGTTGAGATCCAGTTCGATGTAACCCACTCGTGCACCCAACTGATCTTCAGCATCTTTTACTTTCACCAGCGTTTCTGGGTGAGCAAAAACAGGAAGGCAAAATGCCGCAAAAAAGGGAATAAGGGCGACACGGAAATGTTGAATACTCATACTCTTCCTTTTTCAATATTATTGAAGCATTTATCAGGGTTATTGTCTCATGAGCGGATACATATTTGAATGTATTTAGAAAAATAAACAAATAGGGGTTCCGCGCACATTTCCCCGAAAAGTGCCA |
| ***S. aureus* FMN Riboswitch Luciferase construct** | TCACTCATTAGGCACCCCAGGCTTTACACTTTATGCTT**CCGGCTCGTATAATGTGTGCTTTATTCATCTTCGGGGTCGGGTGTAATTCCCAACCGGCAGTAAATAAAGCCTGCGACCTGCTAGTATGTATCATATTAGTGGCTGATCTAGTGAGATTCTAGAGCCGACAGTATAGTCTGGATGGGAGAAGATG**GAGGTTTTTTGTTGTGCAATAATCCTCCTATTCTTACGAGATGAATGGAAGGAGAAAATTGAATATGAATCAGACGCTACTTTGGATCCAAGCAGAGTTGGAAGACGCCAAAAACATAAAGAAAGGCCCGGCGCCATTCTATCCTCTAGAGGATGGAACCGCTGGAGAGCAACTGCATAAGGCTATGAAGAGATACGCCCTGGTTCCTGGAACAATTGCTTTTACAGATGCACATATCGAGGTGAACATCACGTACGCGGAATACTTCGAAATGTCCGTTCGGTTGGCAGAAGCTATGAAACGATATGGGCTGAATACAAATCACAGAATCGTCGTATGCAGTGAAAACTCTCTTCAATTCTTTATGCCGGTGTTGGGCGCGTTATTTATCGGAGTTGCAGTTGCGCCCGCGAACGACATTTATAATGAACGTGAATTGCTCAACAGTATGAACATTTCGCAGCCTACCGTAGTGTTTGTTTCCAAAAAGGGGTTGCAAAAAATTTTGAACGTGCAAAAAAAATTACCAATAATCCAGAAAATTATTATCATGGATTCTAAAACGGATTACCAGGGATTTCAGTCGATGTACACGTTCGTCACATCTCATCTACCTCCCGGTTTTAATGAATACGATTTTGTACCAGAGTCCTTTGATCGTGACAAAACAATTGCACTGATAATGAATTCCTCTGGATCTACTGGGTTACCTAAGGGTGTGGCCCTTCCGCATAGAACTGCCTGCGTCAGATTCTCGCATGCCAGAGATCCTATTTTTGGCAATCAAATCATTCCGGATACTGCGATTTTAAGTGTTGTTCCATTCCATCACGGTTTTGGAATGTTTACTACACTCGGATATTTGATATGTGGATTTCGAGTCGTCTTAATGTATAGATTTGAAGAAGAGCTGTTTTTACGATCCCTTCAGGATTACAAAATTCAAAGTGCGTTGCTAGTACCAACCCTATTTTCATTCTTCGCCAAAAGCACTCTGATTGACAAATACGATTTATCTAATTTACACGAAATTGCTTCTGGGGGCGCACCTCTTTCGAAAGAAGTCGGGGAAGCGGTTGCAAAACGCTTCCATCTTCCAGGGATACGACAAGGATATGGGCTCACTGAGACTACATCAGCTATTCTGATTACACCCGAGGGGGATGATAAACCGGGCGCGGTCGGTAAAGTTGTTCCATTTTTTGAAGCGAAGGTTGTGGATCTGGATACCGGGAAAACGCTGGGCGTTAATCAGAGAGGCGAATTATGTGTCAGAGGACCTATGATTATGTCCGGTTATGTAAACAATCCGGAAGCGACCAACGCCTTGATTGACAAGGATGGATGGCTACATTCTGGAGACATAGCTTACTGGGACGAAGACGAACACTTCTTCATAGTTGACCGCTTGAAGTCTTTAATTAAATACAAAGGATATCAGGTGGCCCCCGCTGAATTGGAATCGATATTGTTACAACACCCCAACATCTTCGACGCGGGCGTGGCAGGTCTTCCCGACGATGACGCCGGTGAACTTCCCGCCGCCGTTGTTGTTTTGGAGCACGGAAAGACGATGACGGAAAAAGAGATCGTGGATTACGTCGCCAGTCAAGTAACAACCGCGAAAAAGTTGCGCGGAGGAGTTGTGTTTGTGGACGAAGTACCGAAAGGTCTTACCGGAAAACTCGACGCAAGAAAAATCAGAGAGATCCTCATAAAGGCCAAGAAGGGCGGAAAGTCCAAATTGTAACTCG |
| ***E.Faecalis Smk Riboswitch*** | TCACTCATTAGGCACCCCAGGCTTTACACTTTATGCTTCCGGCTCGTATAATGTGTGGGAGCTTTTTTTTTAGATATCTGGTATACTGTTTTTG**TTACAAGTTCCCGAAAGGATTTAGCAAGTAATTGTCGTTACTTACTAAAGATGCCTTGTAACCGAAACTATTTAGGGGGAATAACATTA**TGAATCAGACGCTACTTTGGATCCAAGCAGAGTTGGAAGACGCCAAAAACATAAAGAAAGGCCCGGCGCCATTCTATCCTCTAGAGGATGGAACCGCTGGAGAGCAACTGCATAAGGCTATGAAGAGATACGCCCTGGTTCCTGGAACAATTGCTTTTACAGATGCACATATCGAGGTGAACATCACGTACGCGGAATACTTCGAAATGTCCGTTCGGTTGGCAGAAGCTATGAAACGATATGGGCTGAATACAAATCACAGAATCGTCGTATGCAGTGAAAACTCTCTTCAATTCTTTATGCCGGTGTTGGGCGCGTTATTTATCGGAGTTGCAGTTGCGCCCGCGAACGACATTTATAATGAACGTGAATTGCTCAACAGTATGAACATTTCGCAGCCTACCGTAGTGTTTGTTTCCAAAAAGGGGTTGCAAAAAATTTTGAACGTGCAAAAAAAATTACCAATAATCCAGAAAATTATTATCATGGATTCTAAAACGGATTACCAGGGATTTCAGTCGATGTACACGTTCGTCACATCTCATCTACCTCCCGGTTTTAATGAATACGATTTTGTACCAGAGTCCTTTGATCGTGACAAAACAATTGCACTGATAATGAATTCCTCTGGATCTACTGGGTTACCTAAGGGTGTGGCCCTTCCGCATAGAACTGCCTGCGTCAGATTCTCGCATGCCAGAGATCCTATTTTTGGCAATCAAATCATTCCGGATACTGCGATTTTAAGTGTTGTTCCATTCCATCACGGTTTTGGAATGTTTACTACACTCGGATATTTGATATGTGGATTTCGAGTCGTCTTAATGTATAGATTTGAAGAAGAGCTGTTTTTACGATCCCTTCAGGATTACAAAATTCAAAGTGCGTTGCTAGTACCAACCCTATTTTCATTCTTCGCCAAAAGCACTCTGATTGACAAATACGATTTATCTAATTTACACGAAATTGCTTCTGGGGGCGCACCTCTTTCGAAAGAAGTCGGGGAAGCGGTTGCAAAACGCTTCCATCTTCCAGGGATACGACAAGGATATGGGCTCACTGAGACTACATCAGCTATTCTGATTACACCCGAGGGGGATGATAAACCGGGCGCGGTCGGTAAAGTTGTTCCATTTTTTGAAGCGAAGGTTGTGGATCTGGATACCGGGAAAACGCTGGGCGTTAATCAGAGAGGCGAATTATGTGTCAGAGGACCTATGATTATGTCCGGTTATGTAAACAATCCGGAAGCGACCAACGCCTTGATTGACAAGGATGGATGGCTACATTCTGGAGACATAGCTTACTGGGACGAAGACGAACACTTCTTCATAGTTGACCGCTTGAAGTCTTTAATTAAATACAAAGGATATCAGGTGGCCCCCGCTGAATTGGAATCGATATTGTTACAACACCCCAACATCTTCGACGCGGGCGTGGCAGGTCTTCCCGACGATGACGCCGGTGAACTTCCCGCCGCCGTTGTTGTTTTGGAGCACGGAAAGACGATGACGGAAAAAGAGATCGTGGATTACGTCGCCAGTCAAGTAACAACCGCGAAAAAGTTGCGCGGAGGAGTTGTGTTTGTGGACGAAGTACCGAAAGGTCTTACCGGAAAACTCGACGCAAGAAAAATCAGAGAGATCCTCATAAAGGCCAAGAAGGGCGGAAAGTCCAAATTGTAACTCG |
| ***B.subtillis xpt Guanine Riboswitch*** | GCGGAATTCTAATACGACTCACTATAGGGCCTTCGGGCCAAGATCAACGGAACAC**TCATATAATCGCGTGGATATGGCACGCAAGTTTCTACCGGGCACCGTAAATGTCCGACTATGG**GTGAGCAATGGAACCGCACGTGTACGGTTTTTTGTGATATCAGCATTGCTTGCTCTTTATTTGAGCGGGCAATGCTTTTTTTAAATTTATCGTCACGGTGCATCTACCATC |
| ***E.coli ThiC Riboswitch*** | **TAATTTCTTGTCGGAGTGCCTTAACTGGCTGAGACCGTTTATTCGGGATCCGCGGAACCTGATCAGGCTAATACCTGCGAAGGGAACAAGAGTTAATCTGCTATCGCATCGCCTTTGCGGCGATCGTCTCTTGCTTCATCCGTCGTCTGACAAGCCACGTCCTTAACTTTTTGGAATGAGCTATGTCTGCAACAAAACTGA** |

**Table 2: Riboswitch Constructs**

| **Construct Name** | **Sequence *T7 Promotor in italics, Aptamer sequence in italics.** |
| --- | --- |
| ***B. subtillis* FMN Riboswitch** | **TAATACGACTCACTATAGG***GGGCCTTCGGGCCAATGTATCCTTCGGGGCAGGGTGGAAATCCCGACCGGCGGTAGTAAAGCACATTTGCTTTAGAGCCCGTGACCCGTGTGCATAAGCACGCGGTGGATTCAGTTTAAGCTGAAGCCGACAGTGAAAGTCTGGATGGGAGAAGGATGA*TCGATCCGGTTCGCCGGATCCAAATCGGGCTTCGGTCCGGTTC |
| ***F. nucleatum* FMN Riboswitch** | **TAATACGACTCACTATAGG***GGGATCTTCGGGGCAGGGTGAAATTCCCGACCGGTGGTATAGTCCACGAAAGTATTTGCTTTGATTTGGTGAAATTCCAAAACCGACAGTAGAGTCTGGATGAGAGAAGATTCTTTTTGAC*TCGATCCGGTTCGCCGGATCCAAATCGGGCTTCGGTCCGG |
| ***S. aureus* FMN Riboswitch** | **TAATACGACTCACTATAGG***CCGGCTCGTATAATGTGTGCTTTATTCATCTTCGGGGTCGGGTGTAATTCCCAACCGGCAGTAAATAAAGCCTGCGACCTGCTAGTATGTATCATATTAGTGGCTGATCTAGTGAGATTCTAGAGCCGACAGTATAGTCTGGATGGGAGAAGATG*  TCGATCCGGTTCGCCGGATCCAAATCGGGCTTCGGTCCGG |
| ***E. coli FMN Riboswitch*** | **TAATACGACTCACTATAGG***GCTTATTCTCAGGGCGGGGCGAAATTCCCCACCGGCGGTAAATCAACTCAGTTGAAAGCCCGCGAGCGCTTTGGGTGCGAACTCAAAGGACAGCAGATCCGGTGTAATTCCGGGGCCGACGGTTAGAGTCCGGATGGGAGAGAGTAACGAC*TCGATCCGGTTCGCCGGATCCAAATCGGGCTTCGGTCCGG |
| ***E.coli ThiC***  ***Riboswitch*** | **TAATACGACTCACTATAGGG***TAATTTCTTGTCGGAGTGCCTTAACTGGCTGAGACCGTTTATTCGGGATCCGCGGAACCTGATCAGGCTAATACCTGCGAAGGGAACAAGAGTTAATCTGCTATCGCATCGCCCCTGCGGCGATCGTCTCTTGCTTCATCCGTCGTCTGACAAGCCACGTCCTTAACTTTTTGGAATGAGCTATGTCTGCAACAGAC*TCGATCCGGTTCGCCGGATCCAAATCGGGCTTCGGTCCGG |
| ***E.Faecalis Smk Riboswitch*** | **TAATACGACTCACTATAGGG***TTACAAGTTCCCGAAAGGATTTAGCAAGTAATTGTCGTTACTTACTAAAGATGCCTTGTAACCGAAACTATTTAGGGGGAATAACATTATGAATGAC*TCGATCCGGTTCGCCGGATCCAAATCGGGCTTCGGTCCGG |
| ***B.subtillis xpt Guanine Riboswitch*** | **TAATACGACTCACTATAGGGG***GATCATATAATCGCGTGGATATGGCACGCAAGTTTCTACCGGGCACCGTAAATGTCCGACTATGG*TCGACTCGATCCGGTTCGCCGGATCCAAATCGGGCTTCGGTCCGG |

**Table 3: Primers**

| **Primer Name** | **Sequence *Overhangs in bold** |
| --- | --- |
| ***F. nucleatum* Construct Top Primer** | TAATACGACTCACTATAGGGGGATCTTCGGGGCAG |
| ***F. nucleatum* Construct Bottom Primer** | CCGGACCGAAGCCCGATTTGGATCCG |
| ***F. nucleatum* FMN Top Illumina^®^ Overhang** | **AGACGTGTGCTCTTCCGATCT**GGGGATCTTCGGGGCAGGG |
| ***E. coli FMN Construct* Top Overhang** | **TAATACGACTCACTATAGG**GTGCTTATTCTCAGGGCGGGGCGA |
| ***E. coli FMN* Construct Bottom Overhang** | **CCGGACCGAAGCCCGATTTGGATCCGGCGAACCGGATCGAGTC**GTTACTCT CTC CCA |
| ***E. coli* Illumina^®^ Top Overhang** | **AGACGTGTGCTCTCCCGATCT**TGCTTATTCTCAGGGCGGGGCG |
| ***S. aureus Construct* Top Overhang** | **TAATACGACTCACTATAGG**CCGGCTCGTATAATGTGTGCTTT |
| ***S. aureus Construct* Bottom Overhang** | **CCG GAC CGAAGC CCG ATT TGGATCCGGCGA ACC GGATCGA**CATCTTCTCCCATCCAG |
| ***S. aureus* Illumina^®^ Top Overhang** | **AGACGTGTG CTCTTCCGATCT**CCGGCTCGTATAATGTGTGCTT |
| ***B. subtillis* Illumina^®^ Top primer** | **AGACGTGTGCTCTTCCGATCT**CCTTCGGGCCAATGTATCCTTCG |
| **FMN General Illumina^®^ In-Vitro Probing RT primer** | **CTACACGACGCTCTTCCGATCT**CCGGACCGAAGCCCGATTTGGATCC |
| ***B. subtillis* Total RNA FMN RT-Primer** | CCATTTGTTTCCCTCCCCTCTTTTGTTATTTACC |
| ***B. subtillis* Total RNA FMN Illumina^®^ Top Overhang** | **CTACACGACGCTCTTCCGATCT**TCCCCTCTTTTGTTATTTACCGTCAAAAAAGCC |
| ***B. subtillis* Total RNA FMN Illumina^®^ Bottom Overhang** | **AGACGTGTGCTCTTCCGATCT**TGTATCCTTCGGGGCAGGGTGGAAAC |
| ***E.Faecalis Smk Riboswitch Construct Top*** | **TAATACGACTCACTATAGG**GTTACAAGTTCCCGAAAGGATTTA |
| ***E.Faecalis Smk Riboswitch Construct Bottom*** | **CCGGACCGAAGCCCGATTTGGATCCGGCGAACCGGATCGA**GTCATTCATAATGTTATT |
| ***E.Faecalis Smk Riboswitch Illlumina Top*** | **AGACGTGTGCTCTTCCGATCT**GTTACAAGTTCCCGAAAGGATTTAGCAAG |
| ***B.subtillis xpt Guanine Riboswitch Construct Top*** | **TAATACGACTCACTATAGG**GGGATCATATAATCGCGTGGATATGGC |
| ***B.subtillis xpt Guanine Riboswitch Construct Bottom*** | **CCGGACCGAAGCCCGATTTGGATCCGGCGAACCGGATCGA**GTCGACCATAGTCGGA |
| ***B.subtillis xpt Guanine Riboswitch Illumina Top*** | **AGACGTGTGCTCTTCCGATCT**GGATCATATAATCGCGTGGATATGGCAC |
| ***E.coli ThiC***  ***Riboswitch*** | **TAATACGACTCACTATAGG**GTAATTTCTTGTCGGAGTG |
| ***E.coli ThiC***  ***Riboswitch*** | **CCGGACCGAAGCCCGATTTGGATCCGGCGAACCGGATCGA**GTCTGTTGCAGACATAGCTC |
| ***E.coli ThiC***  ***Riboswitch*** | **AGACGTGTGCTCTTCCGATCT**TAATTTCTTGTCGGAGTGCCTTAACTGG |
| ***28S Segment Top Illumina*** | **CTACACGACGCTCTTCCGATCT** AGAAGGGCAAAAGCTCGCTTGA |
| ***28S Segment Bottom***  ***Illumina*** | **AGACGTGTGCTCTTCCGATCT** GCTATGAACGCTTGGCCGCCA |

**Table 4: Split Riboswitch RNA**

| **RNA Name** | **Sequence** |
| --- | --- |
| ***F. nucleatum* FMN aptamer strand 1** | GGAUCUUCGGGGCAGGGUGAAAUUCCCGACCGGUGGUAUAGUCCACGAAAGCUU |
| ***F. nucleatum* FMN aptamer strand 2** | GCUUUGAUUUGGUGAAAUUCCAAAACCGACAGUAGAGUCUGGAUGAGAGAAGAUUC |

**General Synthesis**

Temperatures are given in degrees Celsius (^o^C). Unless stated otherwise, reactions were carried out at room temperature (18-25 ^o^C). Progress of reactions were determined using thin-layer chromatography (TLC) silica gel-coated plates (Merck 60 F254) using the described eluent. Column chromatography was performed with VWR chemicals silica gel (0.040-0.063 mm, pore size 60 Å). The separation was visualized with UV light and/or by potassium permanganate or ninhydrin staining. Chiral separation was achieved using chiral column purchased by Daicel™ (CHIRALFLASH (TM) IA 100 x 30 mm 20 µm Preparative Column). Low resolution electron-spray ionization mass spectra (MS ESI) were recorded on a Single-Quad Thermo ISQ mass spectrometer. NMR spectra were recorded in the indicated solvent at 25 °C on a JEOL 500 MHz spectrometer equipped with a Royal HFX probe. Chemical shifts are displayed in parts per million (ppm) with respect to TMS (δ = 0.00 ppm), CDCl_3_ (δ = 7.26 ppm) or other indicated deuterated solvent as internal reference for ^1^H NMR; and CDCl_3_ (δ = 77.16 ppm) or other indicated deuterated solvent as internal reference for ^13^C NMR. Coupling constants are reported in hertz (Hz) as *J* values. The multiplicity is described by the number of coupling constants, the number of peaks, and the pattern in the signal (s=singlet, d=doublet, t=triplet, q=quartet, dd= doublet of doublets, dt=doublet of triplets, ddd=doublet of doublet of doublets, m=multiplet, br=broad). The peak assignments in ^1^H and ^13^C spectra are based on 2D COSY, HSQC and HMBC spectra. Each stated ^1^H NMR, ^13^C NMR and mass spectra result is depicted at the end of the document.

**Scheme S1:** Synthesis of compound **(-)10**.

(*Z*)-3-amino-3-(thiophen-2-yl)acrylamide (**8**)

Compound **8** was synthesized according to a literature protocol.^10^ In brief, ethyl 3-Oxo-3-(2-thienyl)propionate, **7** (4.6 g, 22.7 mmol, 1.0 eq.) was added to a high pressure tube and was dissolved in 25% NH_4_OH (32 mL). The reaction was stirred at 120 ^o^C for 6 hours. It was then cooled to room temperature and the solid precipitate was collected, washed with H_2_O and dried under reduced pressure to give **8** as an off-white solid (1948 mg, 51% crude yield). The product was used for the next step without any further purification.

**^1^H NMR** (500 MHz, DMSO-*D6*) δ 7.54 (dd, J = 5.0, 1.2 Hz, 1H), 7.45 (dd, J = 3.7, 1.2 Hz, 1H), 7.08 (dd, J = 5.0, 3.7 Hz, 1H), 6.92 – 6.68 (br. s, 2H), 6.29 – 6.04 (br. s, 2H), 4.92 (s, 1H).

*tert*-butyl (*S*)-3-(4-hydroxy-6-(thiophen-2-yl)pyrimidin-2-yl)piperidine-1-carboxylate ((-)**9**)

Compound (-)**9** was synthesized according to a literature protocol.^10^ In brief, Na metal (1449 mg, 63.3 mmol, 15 eq.) was added to a high pressure tube and dissolved in MeOH (20 mL). Once Na metal fully dissolved, **8** (700 mg, 4.16 mmol, 1.0 eq.) was added to the solution along with ethyl 1-(*tert*-butoxycarbonyl)-3-piperidinecarboxylate (2162 mg, 8.40 mmol, 2.0 eq.). The reaction was heated to 100 ^o^C and stirred overnight. The solvent was then removed under reduced pressure and the brown crystals were re-dissolved in H_2_O (30 mL). The solution was neutralized to pH = 7 using aqueous HCl solution and extracted with ethyl acetate (3x). The combined organic layers were washed with brine and dried over MgSO_4_. The crude was purified with column chromatography on silica gel (ethyl acetate : pentane, 1:1 to 4:1). **9** was obtained as a racemic mixture which was further separated with chiral separation (70% heptane : 30% isopropanol : 1% diethylamine) to give (-)**9** as a white solid (660 mg, 43% yield).

**^1^H NMR** (500 MHz, CDCl_3_) δ 7.67 (dd, J = 3.7, 1.2 Hz, 1H), 7.52 – 7.47 (m, 1H), 7.12 (dd, J = 5.0, 3.7 Hz, 1H), 6.62 (s, 1H), 4.36 – 4.15 (m, 1H), 4.07 – 3.98 (m, 1H), 3.30 (d, J = 18.0 Hz, 1H), 2.92 (s, 1H), 2.80 (tt, J = 10.4, 3.9 Hz, 1H), 2.15 (dd, J = 12.4, 4.5 Hz, 1H), 1.94 – 1.79 (m, 2H), 1.69 – 1.57 (m, 1H), 1.45 (s, 9H).

**MS** (ESI) (m/z) calculated for [M+H]^+^:362.46, found 362.29

(*S*)-3-(4-hydroxy-6-(thiophen-2-yl)pyrimidin-2-yl)piperidin-1-ium (**(-)10**)

Compound (-)**10** was synthesized according to a literature protocol.^10^ In brief, (-)**9** (296 mg, 0.819 mmol, 1.0 eq.) was dissolved in ACN/dioxane, 1:4 mixture (3.3 mL). 4M HCl in dioxane (2 mL) was added and the reaction was stirred at room temperature for 6 hours. It was then concentrated under reduced pressure to give (-)**10** as a light brown solid (254 mg, 86% crude yield). The product was used for the next steps without any further purification.

**^1^H NMR** (500 MHz, DMSO-*D_6_*) δ 12.49 (s, 1H), 8.97 (s, 1H), 8.88 (d, J = 10.9 Hz, 1H), 7.84 (dd, J = 3.8, 1.2 Hz, 1H), 7.74 (dd, J = 5.0, 1.1 Hz, 1H), 7.16 (dd, J = 5.0, 3.8 Hz, 1H), 6.69 (s, 1H), 3.44 (dd, J = 12.9, 3.5 Hz, 1H), 3.24 – 3.10 (m, 2H), 3.03 (tt, J = 11.2, 3.7 Hz, 1H), 2.87 (dtdd, J = 13.4, 10.3, 6.7, 2.7 Hz, 1H), 2.08 (dd, J = 12.9, 3.8 Hz, 1H), 1.84 (dt, J = 14.0, 3.7 Hz, 1H), 1.77 – 1.51 (m, 2H).

**MS** (ESI) (m/z) calculated for [M+H]^+^:262.35, found 262.20

**Scheme S2:** Synthesis of compounds **(-)4** - **(-)6**.

methyl (*S*)-6-((3-(4-hydroxy-6-(thiophen-2-yl)pyrimidin-2-yl)piperidin-1-yl)methyl)nicotinate (**(-**

**)6a**)

A flask was dried and put under Argon flow. **(-)10** (50 mg, 0.191 mmol, 1.0 eq.), methyl 6-(bromomethyl)nicotinate (44 mg, 0.191 mmol, 1.0 eq.) and K_2_CO_3_ (26 mg, 0.191 mmol, 1.0 eq.) were added in the flask and suspended in dry ACN (4.8 mL). The reaction was stirred at room temperature overnight. Then, the solvent was removed under reduced pressure and the crude was purified with reverse phase column chromatography (H_2_O with 5% to 50% ACN, 0.1% TFA) to give **(-)6a** as a white solid (52 mg, 65% yield).

**^1^H NMR** (500 MHz, CDCl_3_) δ 9.21 (d, J = 2.1 Hz, 1H), 8.41 (dd, J = 8.0, 2.1 Hz, 1H), 7.76 (d, J = 8.1 Hz, 1H), 7.49 (dd, J = 4.9, 1.1 Hz, 1H), 7.41 – 7.37 (m, 1H), 7.06 (dd, J = 5.1, 3.8 Hz, 1H), 6.42 (s, 1H), 4.60 (d, J = 13.4 Hz, 1H), 4.45 (d, J = 13.4 Hz, 1H), 3.96 (s, 3H), 3.73 – 3.52 (m, 2H), 3.30 (s, 1H), 3.04 (d, J = 13.5 Hz, 1H), 2.35 – 2.26 (m, 1H), 2.15 – 2.00 (m, 2H), 1.65 (s, 1H).

**^13^C NMR** (126 MHz, CDCl_3_) δ 165.00, 164.52, 157.33, 153.05, 150.85, 140.76, 138.99, 136.09, 130.73, 128.59, 127.85, 126.92, 125.97, 104.95, 61.31, 54.44, 52.81, 52.71, 38.33, 27.68, 22.17.

**MS** (ESI) (m/z) calculated for [M+H]^+^:411.49, found 410.88

methyl (*S*)-2-((3-(4-hydroxy-6-(thiophen-2-yl)pyrimidin-2-yl)piperidin-1-yl)methyl)nicotinate (**(-)4a**)

A flask was dried and put under Argon flow. **(-)10** (50 mg, 0.191 mmol, 1.0 eq.), methyl 2-(bromomethyl)nicotinate (44 mg, 0.191 mmol, 1.0 eq.) and K_2_CO_3_ (26 mg, 0.191 mmol, 1.0 eq.) were added in the flask and suspended in dry ACN (4.8 mL). The reaction was stirred at room temperature overnight. Then, the solvent was removed under reduced pressure and the crude was purified with column chromatography on silica gel (DCM : MeOH, 97:3) to give **(-)4a** as a yellow oil (56 mg, 71% yield).

**^1^H NMR** (500 MHz, CDCl_3_) δ 9.09 (dd, J = 4.9, 1.8 Hz, 1H), 8.28 (dd, J = 7.9, 1.8 Hz, 1H), 7.60 (dd, J = 3.7, 1.1 Hz, 1H), 7.42 (dd, J = 5.0, 1.1 Hz, 1H), 7.31 (dd, J = 7.9, 4.8 Hz, 1H), 7.08 (dd, J = 5.0, 3.8 Hz, 1H), 6.58 (s, 1H), 4.16 (d, J = 17.0 Hz, 1H), 4.07 (d, J = 16.9 Hz, 1H), 3.91 (s, 3H), 3.33 (d, J = 12.2 Hz, 1H), 3.03 (dp, J = 7.5, 3.3 Hz, 2H), 2.46 (td, J = 11.2, 3.1 Hz, 1H), 2.36 (dd, J = 12.3, 2.9 Hz, 1H), 2.28 (d, J = 13.3 Hz, 1H), 1.82 – 1.55 (m, 3H).

**^13^C NMR** (126 MHz, CDCl_3_) δ 166.25, 165.00, 163.96, 159.01, 156.96, 152.56, 142.50, 139.34, 129.29, 128.30, 126.67, 124.60, 122.09, 105.07, 62.23, 55.24, 53.76, 52.52, 39.63, 29.48, 22.57.

**MS** (ESI) (m/z) calculated for [M+H]^+^:411.49, found 411.11

methyl (**S**)-5-((3-(4-hydroxy-6-(thiophen-2-yl)pyrimidin-2-yl)piperidin-1-yl)methyl)nicotinate (**(-)5a**)

A flask was dried and put under Argon flow. **(-)10** (30 mg, 0.101 mmol, 1.0 eq.), methyl 5-(chloromethyl)nicotinate hydrochloride (46 mg, 0.207 mmol, 2.0 eq.) and K_2_CO_3_ (61 mg, 0.442 mmol, 4.4 eq.) were added in the flask and suspended in dry ACN (2.9 mL). The reaction was stirred at room temperature overnight. Then, the solvent was removed under reduced pressure and the crude was purified with column chromatography on silica gel (DCM : MeOH, 95:5 to 93:7) to give **(-)5a** as a white solid (26 mg, 63% yield).

**^1^H NMR** (500 MHz, CDCl_3_) δ 9.10 (d, J = 1.9 Hz, 1H), 8.75 (d, J = 2.3 Hz, 1H), 8.30 (t, J = 2.1 Hz, 1H), 7.63 (d, J = 1.2 Hz, 1H), 7.46 (dd, J = 5.0, 1.1 Hz, 1H), 7.10 (dd, J = 5.0, 3.7 Hz, 1H), 6.57 (s, 1H), 3.94 (s, 3H), 3.70 – 3.60 (m, 2H), 3.05 – 2.97 (m, 1H), 2.87 – 2.69 (m, 2H), 2.67 – 2.57 (m, 1H), 2.54 – 2.40 (m, 1H), 1.90 (q, J = 6.2 Hz, 2H), 1.83 – 1.65 (m, 2H).

**^13^C NMR** (126 MHz, CDCl_3_) δ 165.78, 163.72, 157.45, 154.14, 150.11, 142.01, 137.85, 132.78, 129.90, 128.45, 127.21, 126.14, 105.04, 60.10, 55.56, 52.55, 50.86, 40.10, 29.77, 28.29, 22.93.

**MS** (ESI) (m/z) calculated for [M+H]^+^: 411.49, found 411.21

(*S*)-6-((3-(4-hydroxy-6-(thiophen-2-yl)pyrimidin-2-yl)piperidin-1-yl)methyl)nicotinic acid (**(-)6b**)

**(-)6a** (52 mg, 0.127 mmol) was suspended in MeOH (5.1 mL) and 4M NaOH aqueous solution (3 mL) was added. The reaction was stirred at room temperature for 4 hours and it was subsequently neutralized with an aqueous HCl solution. It was then purified by reverse phase column chromatography (H_2_O with 5% to 100% ACN, 0.1% TFA) to give **(-)6b** as a white solid (34 mg, 67% yield).

**^1^H NMR** (500 MHz, DMSO-*D_6_*) δ 9.13 (d, J = 2.2 Hz, 1H), 8.39 (dd, J = 8.1, 2.2 Hz, 1H), 7.87 (dd, J = 3.8, 1.2 Hz, 1H), 7.78 (dd, J = 5.0, 1.1 Hz, 1H), 7.71 (d, J = 8.1 Hz, 1H), 7.19 (dd, J = 5.0, 3.7 Hz, 1H), 6.71 (s, 1H), 4.63 (s, 2H), 3.73 – 3.30 (m, 2H), 3.26 – 3.04 (m, 2H), 2.15 – 2.08 (m, 1H), 1.97 – 1.85 (m, 2H), 1.73 – 1.53 (m, 1H).

**^13^C NMR** (126 MHz, DMSO-*D_6_*) δ 166.31, 158.61, 155.94, 155.26, 150.63, 141.83, 138.88, 131.08, 129.35, 128.20, 127.23, 125.30, 118.90, 116.51, 105.39, 60.43, 53.58, 52.77, 27.77, 22.37.

**HRMS** (ESI) (m/z) calculated for [M+H]^+^:397.1329, found 397.1339

(*S*)-2-((3-(4-hydroxy-6-(thiophen-2-yl)pyrimidin-2-yl)piperidin-1-yl)methyl)nicotinic acid (**(-)4b**)

Compound **(-)4a** (55 mg, 0.134 mmol) was suspended in MeOH (5.4 mL) and 4M NaOH aqueous solution (3.2 mL) was added. The reaction was stirred at room temperature for 4 hours and it was subsequently neutralized with an aqueous HCl solution. It was then purified by reverse phase column chromatography (H_2_O with 5% to 100% ACN, 0.1% TFA) to give **(-)4b** as a white solid (41 mg, 77% yield).

**^1^H NMR** (500 MHz, DMSO-*D_6_*) δ 8.76 (s, 1H), 8.37 (dd, J = 7.9, 1.7 Hz, 1H), 7.85 (d, J = 3.8 Hz, 1H), 7.73 (dd, J = 5.0, 1.2 Hz, 1H), 7.59 (dd, J = 7.9, 4.9 Hz, 1H), 7.21 – 7.09 (m, 1H), 6.67 (s, 1H), 4.93 (s, 2H), 3.75 – 3.66 (m, 1H), 3.61 – 3.39 (m, 1H), 3.29 – 3.25 (m, 3H), 2.13 – 2.07 (m, 1H), 1.93 (pt, J = 13.4, 7.1 Hz, 2H), 1.73 – 1.55 (m, 1H).

**^13^C NMR** (126 MHz, DMSO-*D_6_*) δ 167.07, 158.66, 158.40, 152.11, 151.75, 141.69, 140.21, 131.13, 129.29, 128.33, 126.79, 124.42, 118.77, 116.40, 105.54, 59.93, 53.95, 53.10, 36.31, 31.30.

**HRMS** (ESI) (m/z) calculated for [M+H]^+^:397.1329, found 397.1339

(*S*)-5-((3-(4-hydroxy-6-(thiophen-2-yl)pyrimidin-2-yl)piperidin-1-yl)methyl)nicotinic acid (**(-)5b**)

Compound **(-)5a** (26 mg, 0.063 mmol) was suspended in MeOH (2.5 mL) and 4M NaOH aqueous solution (1.5 mL) was added. The reaction was stirred at room temperature for 4 hours and it was subsequently neutralized with an aqueous HCl solution. It was then purified by reverse phase column chromatography (H_2_O with 5% to 100% ACN, 0.1% TFA) to give **(-)5b** as a white solid (25 mg, quant. yield).

**^1^H NMR** (500 MHz, DMSO-*D_6_*) δ 9.10 (d, J = 2.0 Hz, 1H), 8.88 (d, J = 2.3 Hz, 1H), 8.49 (s, 1H), 7.83 (d, J = 3.8 Hz, 1H), 7.74 (d, J = 5.0 Hz, 1H), 7.16 (dd, J = 5.0, 3.8 Hz, 1H), 6.67 (s, 1H), 4.53 (q, J = 13.1 Hz, 2H), 3.66 – 3.52 (m, 1H), 3.49 – 3.40 (m, 1H), 3.30 – 3.16 (m, 1H), 3.10 – 2.91 (m, 2H), 2.17 – 2.08 (m, 1H), 1.99 – 1.88 (m, 1H), 1.81 – 1.65 (m, 1H), 1.59 – 1.44 (m, 1H).

**^13^C NMR** (126 MHz, DMSO-*D_6_*) δ 166.48, 156.10, 151.65, 141.91, 140.67, 131.10, 129.34, 128.13, 127.22, 126.27, 120.51, 118.16, 115.80, 113.45, 105.39, 57.01, 52.57, 52.11, 38.43, 27.58, 22.74.

**HRMS** (ESI) (m/z) calculated for [M+H]^+^:397.1329, found 398.1338

(*S*)-(6-((3-(4-hydroxy-6-(thiophen-2-yl)pyrimidin-2-yl)piperidin-1-yl)methyl)pyridin-3-yl)(1*H*-imidazol-1-yl)methanone (**(-)6**)

Compound **(-)6b** (2.8 mg, 0.007 mmol, 1.0 eq.) and 1,1′-carbonyldiimidazole (2.3 mg, 0.013 mmol, 2.0 eq.) were dissolved in dry DMSO-d6 (0.5 mL). The solution was briefly vortexed, and the reaction was allowed to proceed at room temperature for 15 minutes.

**^1^H NMR** (500 MHz, DMSO-*D*_6_) δ 8.89 (d, *J* = 2.0 Hz, 1H), 8.25 – 8.21 (m, 1H), 8.17 (dt, *J* = 8.1, 1.9 Hz, 1H), 7.83 – 7.78 (m, 1H), 7.70 (dd, *J* = 4.5, 2.8 Hz, 2H), 7.64 (d, *J* = 8.1 Hz, 1H), 7.14 (q, *J* = 1.3 Hz, 2H), 6.63 (d, *J* = 1.6 Hz, 1H), 3.84 – 3.72 (m, 2H), 2.98 – 2.92 (m, 1H), 2.90 – 2.75 (m, 2H), 2.51 (d, *J* = 10.8 Hz, 1H), 2.29 – 2.20 (m, 1H), 1.89 (td, *J* = 10.2, 3.8 Hz, 1H), 1.76 – 1.67 (m, 1H), 1.67 – 1.47 (m, 2H).

(*S*)-(2-((3-(4-hydroxy-6-(thiophen-2-yl)pyrimidin-2-yl)piperidin-1-yl)methyl)pyridin-3-yl)(1*H*-imidazol-1-yl)methanone (**(-)4**)

Compound **(-)4b** (3.0 mg, 0.008 mmol, 1.0 eq.) and 1,1′-carbonyldiimidazole (2.5 mg, 0.015 mmol, 2.0 eq.) were dissolved in dry DMSO-d6 (0.5 mL). The solution was briefly vortexed, and the reaction was allowed to proceed at room temperature for 15 minutes.

**^1^H NMR** (500 MHz, DMSO-*D*_6_) δ 8.63 (dd, *J* = 4.9, 1.7 Hz, 1H), 8.14 (s, 1H), 7.97 (dd, *J* = 7.7, 1.7 Hz, 1H), 7.78 (dd, *J* = 3.7, 1.2 Hz, 1H), 7.69 (dd, *J* = 5.0, 1.1 Hz, 1H), 7.65 (s, 1H), 7.46 (dd, *J* = 7.7, 4.8 Hz, 1H), 7.12 (d, *J* = 1.3 Hz, 2H), 6.59 (s, 1H), 3.68 (q, *J* = 14.6 Hz, 2H), 2.60 (dq, *J* = 6.0, 2.1 Hz, 1H), 2.39 (d, *J* = 11.5 Hz, 1H), 2.27 – 2.17 (m, 2H), 1.99 – 1.88 (m, 1H), 1.76 – 1.66 (m, 1H), 1.42 (dt, *J* = 12.9, 3.3 Hz, 1H), 1.29 (qd, *J* = 11.6, 3.0 Hz, 1H), 0.80 (tt, *J* = 13.3, 2.8 Hz, 1H).

(*S*)-(5-((3-(4-hydroxy-6-(thiophen-2-yl)pyrimidin-2-yl)piperidin-1-yl)methyl)pyridin-3-yl)(1*H*-imidazol-1-yl)methanone (**(-)5**)

Compound **(-)5b** (3.0 mg, 0.008 mmol, 1.0 eq.) and 1,1′-carbonyldiimidazole (2.4 mg, 0.015 mmol, 2.0 eq.) were dissolved in dry DMSO-d6 (0.5 mL). The solution was briefly vortexed, and the reaction was allowed to proceed at room temperature for 15 minutes.

**^1^H NMR** (500 MHz, DMSO-*D*_6_) δ 8.85 (d, *J* = 2.1 Hz, 1H), 8.79 (d, *J* = 2.0 Hz, 1H), 8.23 (t, *J* = 1.1 Hz, 1H), 8.10 (t, *J* = 2.1 Hz, 1H), 7.79 (dd, *J* = 3.7, 1.2 Hz, 1H), 7.70 – 7.68 (m, 2H), 7.14 – 7.12 (m, 2H), 6.61 (s, 1H), 3.77 – 3.67 (m, 2H), 3.02 – 2.97 (m, 1H), 2.87 – 2.74 (m, 2H), 2.39 – 2.31 (m, 1H), 2.13 (t, *J* = 10.1 Hz, 1H), 1.90 (d, *J* = 7.8 Hz, 1H), 1.75 – 1.69 (m, 1H), 1.59 – 1.49 (m, 2H).

**Scheme S3:** Synthesis of compounds **(-)1** - **(-)3**.

(*S*)-2-(1-(prop-2-yn-1-yl)piperidin-3-yl)-6-(thiophen-2-yl)pyrimidin-4-ol (**(-)11**)

A flask was dried and put under Argon flow. **(-)10** (200 mg, 0.674 mmol, 1.0 eq.), propargyl bromide, 80 wt% solution in toluene (0.08 mL, 0.674 mmol, 1.0 eq.) and K_2_CO_3_ (94 mg, 0.674 mmol, 1.0 eq.) were added in the flask and suspended in dry ACN (16.8 mL). The reaction was stirred at room temperature overnight. Then, the solvent was removed under reduced pressure and the crude was purified with column chromatography on silica gel (DCM : MeOH, 9:1) to give **(-)11** as a white solid (121 mg, 60% yield).

**^1^H NMR** (500 MHz, CDCl_3_) δ 7.62 (dd, J = 3.8, 1.2 Hz, 1H), 7.46 (dd, J = 5.0, 1.1 Hz, 1H), 7.15 – 7.07 (m, 1H), 6.59 (s, 1H), 3.48 (s, 4H), 3.43 (dd, J = 9.4, 2.4 Hz, 2H), 3.15 – 3.00 (m, 2H), 2.88 – 2.81 (m, 1H), 2.76 (dd, J = 12.2, 3.4 Hz, 1H), 2.54 – 2.45 (m, 1H), 2.30 (t, J = 2.4 Hz, 1H), 2.05 – 1.95 (m, 1H), 1.86 – 1.71 (m, 1H).

**^13^C NMR** (126 MHz, CDCl_3_) δ 164.54, 162.96, 157.38, 141.95, 129.80, 128.45, 127.08, 105.28, 74.55, 53.51, 52.26, 50.97, 47.28, 39.07, 27.99, 21.84.

**MS** (ESI) (m/z) calculated for [M+H]^+^:300.39, found 300.26

methyl (*S*)-4-(4-((3-(4-hydroxy-6-(thiophen-2-yl)pyrimidin-2-yl)piperidin-1-yl)methyl)-1H-1,2,3-triazol-1-yl)benzoate (**(-)3a**)

Compound **(-)11** (30 mg, 0.100 mmol, 1.0 eq.) and **13** (33 mg, 0.200 mmol, 2.0 eq.) were added in a flask and they were suspended in DMF/H_2_O, 1:1 mixture (0.11 mL). Sodium ascorbate (2.0 mg, 0.01 mmol, 0.1 eq.) and CuSO_4_.5H_2_O (1.2 mg, 0.005 mmol, 0.05 eq.) were added and the reaction was stirred at room temperature overnight. It was then diluted with H_2_O and the solid precipitate was collected. It was washed once more with H_2_O and freeze dried. Next, it was purified by column chromatography (DCM : MeOH, 9:1) to give **(-)3a** as a white solid (22 mg, 46% yield).

**^1^H NMR** (500 MHz, CDCl_3_) δ 8.64 (s, 1H), 8.23 – 8.15 (m, 2H), 8.02 – 7.96 (m, 2H), 7.60 (dd, *J* = 3.8, 1.2 Hz, 1H), 7.45 (dd, *J* = 5.0, 1.1 Hz, 1H), 7.09 (dd, *J* = 5.0, 3.7 Hz, 1H), 6.57 (s, 1H), 3.97 – 3.90 (m, 5H), 3.81 (d, *J* = 13.9 Hz, 1H), 3.19 – 3.03 (m, 3H), 2.59 (d, *J* = 11.7 Hz, 1H), 2.44 (t, *J* = 10.7 Hz, 1H), 2.05 (d, *J* = 12.7 Hz, 1H), 1.89 – 1.67 (m, 3H).

**^13^C NMR** (126 MHz, CDCl_3_) δ 166.11, 164.56, 163.02, 157.76, 145.35, 141.90, 140.17, 131.49, 130.18, 129.87, 128.44, 127.17, 121.33, 119.85, 105.17, 54.49, 54.00, 53.59, 53.19, 52.47, 38.32, 28.10, 21.85.

**HRMS** (ESI) (m/z) calculated for [M+H]^+^:477.1703, found 477.1698

methyl (*S*)-3-(4-((3-(4-hydroxy-6-(thiophen-2-yl)pyrimidin-2-yl)piperidin-1-yl)methyl)-1H-1,2,3-triazol-1-yl)benzoate (**(-)2a**)

**(-)11** (30 mg, 0.100 mmol, 1.0 eq.), methyl 3-azidobenzoate, **14** (35 mg, 0.200 mmol, 2.0 eq.), sodium ascorbate (2.0 mg, 0.01 mmol, 0.1 eq.), and CuSO_4_.5H_2_O (1.2 mg, 0.005 mmol, 0.05 eq.) were suspended in DMF/H_2_O, 1:1 mixture (0.5 mL) and the reaction was stirred at room temperature overnight. It was then diluted with H_2_O and the solid precipitate was collected. Next, it was purified by column chromatography (DCM : MeOH, 96:4) to give **(-)2a** as an off-white solid (43 mg, 90% yield).

**^1^H NMR** (500 MHz, CDCl_3_) δ 8.56 – 8.50 (m, 2H), 8.10 – 8.03 (m, 2H), 7.64 – 7.57 (m, 2H), 7.44 (dd, *J* = 5.0, 1.1 Hz, 1H), 7.08 (dd, *J* = 5.0, 3.7 Hz, 1H), 6.57 (s, 1H), 3.95 (s, 4H), 3.81 (d, *J* = 13.9 Hz, 1H), 3.15 (d, *J* = 12.1 Hz, 1H), 3.06 (dt, *J* = 5.3, 3.1 Hz, 2H), 2.60 (d, *J* = 12.0 Hz, 1H), 2.42 (t, *J* = 11.1 Hz, 1H), 2.00 (d, *J* = 18.7 Hz, 2H), 1.88 – 1.66 (m, 2H).

**^13^C NMR** (126 MHz, CDCl_3_) δ 165.97, 164.59, 163.03, 157.67, 145.14, 141.94, 137.28, 132.02, 130.14, 129.82, 129.67, 128.42, 127.14, 124.54, 121.45, 121.37, 105.21, 54.55, 53.89, 53.55, 52.61, 38.47, 28.15, 21.90.

**MS** (ESI) (m/z) calculated for [M+H]^+^: 477.56, found 477.13

methyl (*S*)-2-(4-((3-(4-hydroxy-6-(thiophen-2-yl)pyrimidin-2-yl)piperidin-1-yl)methyl)-1H-1,2,3-triazol-1-yl)benzoate (**(-)1a**)

**(-)11** (30 mg, 0.100 mmol, 1.0 eq.), methyl 2-azidobenzoate, **15** (35 mg, 0.200 mmol, 2.0 eq.), sodium ascorbate (2.0 mg, 0.01 mmol, 0.1 eq.), and CuSO_4_.5H_2_O (1.2 mg, 0.005 mmol, 0.05 eq.) were suspended in DMF/H_2_O, 1:1 mixture (0.5 mL) and the reaction was stirred at room temperature overnight. It was then diluted with H_2_O and the solid precipitate was collected. Next, it was purified by column chromatography (DCM : MeOH, 98:2 to 96:4) to give **(-)1a** as a yellow oil (42 mg, 89% yield).

**^1^H NMR** (500 MHz, CDCl_3_) δ 8.14 (d, *J* = 5.2 Hz, 1H), 7.95 (ddd, *J* = 7.8, 4.2, 1.5 Hz, 1H), 7.67 – 7.49 (m, 5H), 7.43 (ddd, *J* = 5.1, 1.8, 1.1 Hz, 1H), 7.07 (ddd, *J* = 5.1, 3.8, 2.3 Hz, 1H), 3.92 (dd, *J* = 13.8, 3.3 Hz, 1H), 3.80 (d, *J* = 13.8 Hz, 1H), 3.62 (d, *J* = 2.3 Hz, 3H), 3.21 – 2.91 (m, 3H), 2.70 – 2.33 (m, 2H), 2.00 (dt, *J* = 18.2, 5.0 Hz, 1H), 1.88 – 1.64 (m, 3H).

**^13^C NMR** (126 MHz, CDCl_3_) δ 165.48, 164.59, 163.04, 157.53, 143.69, 141.98, 136.34, 132.88, 131.28, 129.90, 129.78, 128.41, 127.38, 127.09, 126.82, 125.06, 105.06, 54.42, 53.82, 52.49, 38.81, 28.19, 22.11.

**MS** (ESI) (m/z) calculated for [M+H]^+^: 477.56, found 477.12

(*S*)-4-(4-((3-(4-hydroxy-6-(thiophen-2-yl)pyrimidin-2-yl)piperidin-1-yl)methyl)-1H-1,2,3-triazol-1-yl)benzoic acid (**(-)3b**)

Compound **(-)3a** (47 mg, 0.099 mmol) was suspended in MeOH (3.96 mL) and 4M NaOH aqueous solution (2.36 mL) was added. The reaction was stirred at room temperature for 4 hours and it was subsequently neutralized with an aqueous HCl solution. It was then purified by reverse phase column (H_2_O with 5% to 100% ACN, 0.1% TFA) to give **(-)3b** as a white solid (45 mg, 98% yield).

**^1^H NMR** (500 MHz, DMSO-*D*_6_) δ 9.00 (s, 1H), 8.16 – 8.10 (m, 2H), 8.08 – 8.02 (m, 2H), 7.82 (dd, *J* = 3.8, 1.0 Hz, 1H), 7.69 (d, *J* = 5.0 Hz, 1H), 7.14 (dd, *J* = 5.0, 3.8 Hz, 1H), 6.66 (s, 1H), 4.39 (s, 2H), 3.54 (d, *J* = 11.3 Hz, 1H), 3.35 (d, *J* = 11.7 Hz, 1H), 3.05 (d, *J* = 11.1 Hz, 1H), 2.87 (s, 2H), 2.05 – 1.99 (m, 1H), 1.88 (dt, *J* = 14.4, 3.5 Hz, 1H), 1.80 – 1.67 (m, 1H), 1.58 (d, *J* = 12.3 Hz, 1H).

**^13^C NMR** (126 MHz, DMSO-*D*_6_) δ 166.89, 166.06, 156.07, 148.75, 141.91, 139.85, 139.78, 134.93, 131.69, 131.47, 131.06, 129.30, 128.10, 125.37, 120.51, 119.98, 105.20, 53.41, 52.15, 51.25, 39.16, 27.61, 22.83.

**HRMS** (ESI) (m/z) calculated for [M-H]^+^:461.1402, found 461,1404

(S)-3-(4-((3-(4-hydroxy-6-(thiophen-2-yl)pyrimidin-2-yl)piperidin-1-yl)methyl)-1H-1,2,3-triazol-1-yl)benzoic acid (**(-)2b**)

Compound **(-)2a** (48 mg, 0.100 mmol) was suspended in MeOH (4 mL) and 4M NaOH aqueous solution (2.4 mL) was added. The reaction was stirred at room temperature for 4 hours and it was subsequently neutralized with an aqueous HCl solution. It was then purified by reverse phase column (H_2_O with 5% to 100% ACN, 0.1% TFA) to give **(-)2b** as a white solid (31 mg, 65% yield).

**^1^H NMR** (500 MHz, DMSO-*D*_6_) δ 9.07 (s, 1H), 8.40 (t, *J* = 1.9 Hz, 1H), 8.19 – 8.13 (m, 1H), 8.05 (dt, *J* = 7.8, 1.3 Hz, 1H), 7.83 (d, *J* = 3.7 Hz, 1H), 7.79 – 7.69 (m, 2H), 7.14 (q, *J* = 6.4 Hz, 1H), 6.68 (s, 1H), 4.59 (s, 2H), 3.76 – 3.71 (m, 2H), 3.55 (d, *J* = 12.0 Hz, 1H), 3.28 (s, 1H), 3.06 (qd, *J* = 7.2, 4.6 Hz, 1H), 2.07 (d, *J* = 12.2 Hz, 1H), 1.95 (d, *J* = 14.2 Hz, 1H), 1.77 (d, *J* = 14.0 Hz, 1H), 1.54 (d, *J* = 13.0 Hz, 1H).

**^13^C NMR** (126 MHz, DMSO-*D*_6_) δ 166.79, 141.84, 138.01, 137.07, 133.23, 132.79, 131.09, 130.18, 129.27, 128.19, 126.24, 124.99, 121.32, 105.46, 52.76, 51.89, 50.87, 46.27, 38.92, 27.58, 22.60.

**HRMS** (ESI) (m/z) calculated for [M+H]^+^:463.1547, found 463.1558

(*S*)-2-(4-((3-(4-hydroxy-6-(thiophen-2-yl)pyrimidin-2-yl)piperidin-1-yl)methyl)-1H-1,2,3-triazol-1-yl)benzoic acid (**(-)1b**)

Compound **(-)1a** (42 mg, 0.088 mmol) was suspended in MeOH (3.5 mL) and 4M NaOH aqueous solution (2.1 mL) was added. The reaction was stirred at room temperature for 4 hours and it was subsequently neutralized with an aqueous HCl solution. It was then purified by reverse phase column (H_2_O with 5% to 100% ACN, 0.1% TFA) to give **(-)1b** as a white solid (36 mg, 85% yield).

**^1^H NMR** (500 MHz, DMSO-*D*_6_) δ 11.70 (s, 1H), 8.71 (s, 1H), 7.95 (dd, *J* = 7.7, 1.6 Hz, 1H), 7.85 – 7.75 (m, 2H), 7.72 – 7.67 (m, 2H), 7.63 (dd, *J* = 7.8, 1.2 Hz, 1H), 7.13 (dd, *J* = 5.0, 3.7 Hz, 1H), 6.66 (s, 1H), 4.53 (t, *J* = 7.3 Hz, 2H), 3.89 – 3.26 (m, 3H), 2.90 (d, *J* = 13.7 Hz, 1H), 2.17 – 2.07 (m, 1H), 2.02 – 1.84 (m, 3H), 1.49 (tt, *J* = 11.4, 6.3 Hz, 1H).

**^13^C NMR** (126 MHz, DMSO-*D*_6_) δ 166.68, 163.11, 161.95, 156.03, 141.83, 136.78, 135.72, 133.24, 131.30, 131.13, 130.97, 129.50, 129.27, 129.06, 128.16, 127.37, 105.30, 52.37, 51.07, 50.66, 38.48, 27.98, 22.34.

**HRMS** (ESI) (m/z) calculated for [M+H]^+^:463.1547, found 463.1557

(*S*)-(4-(4-((3-(4-hydroxy-6-(thiophen-2-yl)pyrimidin-2-yl)piperidin-1-yl)methyl)-1H-1,2,3-triazol-1-yl)phenyl)(1*H*-imidazol-1-yl)methanone (**(-)3**)

Compound **(-)3b** (7 mg, 0.015 mmol, 1.0 eq.) and 1,1′-carbonyldiimidazole (4.9 mg, 0.030 mmol, 2.0 eq.) were dissolved in dry DMSO-d6 (0.5 mL). The solution was briefly vortexed, and the reaction was allowed to proceed at room temperature for 15 minutes.

**^1^H NMR** (499 MHz, DMSO-*D*_6_) δ 8.64 (td, *J* = 5.0, 2.3 Hz, 1H), 8.18 – 8.05 (m, 4H), 8.00 – 7.94 (m, 1H), 7.78 (dd, *J* = 3.7, 1.3 Hz, 1H), 7.71 – 7.68 (m, 1H), 7.67 – 7.64 (m, 1H), 7.46 (dd, *J* = 7.8, 4.8 Hz, 1H), 7.13 – 7.09 (m, 1H), 6.59 (s, 1H), 3.81 – 3.57 (m, 2H), 2.62 – 2.56 (m, 1H), 2.43 – 2.36 (m, 1H), 2.28 – 2.17 (m, 2H), 1.99 – 1.89 (m, 1H), 1.75 – 1.66 (m, 1H), 1.49 – 1.35 (m, 1H), 1.35 – 1.26 (m, 1H), 0.86 – 0.76 (m, 1H).

(*S*)-(3-(4-((3-(4-hydroxy-6-(thiophen-2-yl)pyrimidin-2-yl)piperidin-1-yl)methyl)-1H-1,2,3-triazol-1-yl)phenyl)(1*H*-imidazol-1-yl)methanone (**(-)2**)

Compound **(-)2b** (2.8 mg, 0.006 mmol, 1.0 eq.) and 1,1′-carbonyldiimidazole (2 mg, 0.001 mmol, 2.0 eq.) were dissolved in dry DMSO-d6 (0.5 mL). The solution was briefly vortexed, and the reaction was allowed to proceed at room temperature for 15 minutes.

**^1^H NMR** (500 MHz, DMSO-*D*_6_) δ 8.84 (s, 1H), 8.31 (t, *J* = 1.9 Hz, 1H), 8.29 – 8.22 (m, 2H), 7.87 (dt, *J* = 7.8, 1.4 Hz, 1H), 7.81 – 7.78 (m, 2H), 7.72 (t, *J* = 1.5 Hz, 1H), 7.69 (dd, *J* = 4.9, 1.1 Hz, 1H), 7.13 (dd, *J* = 5.0, 3.7 Hz, 1H), 6.62 (s, 1H), 3.78 (d, *J* = 2.5 Hz, 2H), 3.10 – 3.02 (m, 1H), 2.94 – 2.87 (m, 1H), 2.79 (ddt, *J* = 10.9, 7.1, 3.6 Hz, 1H), 2.40 (t, *J* = 10.9 Hz, 1H), 2.23 – 2.15 (m, 1H), 1.88 (dp, *J* = 8.2, 4.6 Hz, 1H), 1.73 (dqd, *J* = 9.7, 7.0, 3.3 Hz, 1H), 1.59 – 1.50 (m, 2H).

(S)-(2-(4-((3-(4-hydroxy-6-(thiophen-2-yl)pyrimidin-2-yl)piperidin-1-yl)methyl)-1H-1,2,3-triazol-1-yl)phenyl)(1H-imidazol-1-yl)methanone (**(-)1**)

Compound **(-)1b** (2.9 mg, 0.006 mmol, 1.0 eq.) and 1,1′-carbonyldiimidazole (2 mg, 0.001 mmol, 2.0 eq.) were dissolved in dry DMSO-d6 (0.5 mL). The solution was briefly vortexed, and the reaction was allowed to proceed at room temperature for 15 minutes.

**^1^H NMR** (500 MHz, DMSO-*D*_6_) δ 8.57 (s, 1H), 7.91 (s, 1H), 7.90 – 7.83 (m, 3H), 7.80 (dd, *J* = 3.8, 1.2 Hz, 1H), 7.70 (dd, *J* = 5.0, 1.1 Hz, 2H), 7.40 (t, *J* = 1.6 Hz, 1H), 7.13 (d, *J* = 1.3 Hz, 1H), 6.92 – 6.88 (m, 1H), 6.63 (s, 1H), 3.65 (s, 2H), 2.95 – 2.90 (m, 1H), 2.77 (s, 1H), 2.74 (d, *J* = 10.7 Hz, 1H), 2.36 – 2.23 (m, 1H), 2.11 – 1.97 (m, 1H), 1.93 – 1.81 (m, 1H), 1.75 – 1.63 (m, 1H), 1.53 (dq, *J* = 10.9, 6.3 Hz, 2H).

**Scheme S4:** Synthesis of compound **13**.

methyl 4-azidobenzoate (**13**)

A flask was dried and put under Ar flow. 4-Azidobenzoic acid, **12** (150 mg, 0.920 mmol, 1.0 eq.) was added and it was dissolved in dry MeOH (5.11 mL). The mixture was cooled at 0 ^o^C and SOCl_2_ (0.15 mL, 2.11 mmol, 2.3 eq.) was added dropwise. The reaction was then stirred at room temperature overnight. Afterwards, the solvent was evaporated under vacuum and the residue was purified by column chromatography (ethyl acetate : pentane, 1:2) to give **13** as a light yellow oil (149 mg, 92 % yield).

**^1^H NMR** (500 MHz, CDCl_3_) δ 8.05 – 7.98 (m, 2H), 7.08 – 7.02 (m, 2H), 3.89 (s, 3H).

**^13^C NMR** (126 MHz, CDCl_3_) δ 166.37, 144.83, 131.49, 126.79, 118.92, 52.23

o

**References**

(1)	Howe, J. A.; Xiao ,Li; Fischmann ,Thierry O.; Wang ,Hao; Tang ,Haifeng; Villafania ,Artjohn; Zhang ,Rumin; Barbieri ,Christopher M.; and Roemer, T. Atomic Resolution Mechanistic Studies of Ribocil: A Highly Selective Unnatural Ligand Mimic of the E. Coli FMN Riboswitch. *RNA Biology* **2016**, *13* (10), 946–954. https://doi.org/10.1080/15476286.2016.1216304.

(2)	Li, C.; Yang, X.; Dickerson, K. A.; Zanon, P. R. A.; Springer, N. A.; Wang, J.; Jia, Y.; Munshi, N. C.; Batey, R. T.; Disney, M. D. Structure-Guided Design of a Bioactive Covalent Small Molecule Targeting a Riboswitch. *J. Am. Chem. Soc.* **2025**, *147* (42), 38684–38690. https://doi.org/10.1021/jacs.5c13219.

(3)	Crielaard, S.; Maassen, R.; Vosman, T.; Rempkens, I.; Velema, W. A. Affinity-Based Profiling of the Flavin Mononucleotide Riboswitch. *J Am Chem Soc* **2022**, *144* (23), 10462–10470. https://doi.org/10.1021/jacs.2c02685.

(4)	Pedrolli, D.; Langer, S.; Hobl, B.; Schwarz, J.; Hashimoto, M.; Mack, M. The ribB FMN Riboswitch from Escherichia Coli Operates at the Transcriptional and Translational Level and Regulates Riboflavin Biosynthesis. *FEBS J* **2015**, *282* (16), 3230–3242. https://doi.org/10.1111/febs.13226.

(5)	Spitale, R. C.; Crisalli, P.; Flynn, R. A.; Torre, E. A.; Kool, E. T.; Chang, H. Y. RNA SHAPE Analysis in Living Cells. *Nature Chemical Biology* **2013**, *9* (1), 18–20. https://doi.org/10.1038/nchembio.1131.

(6)	Incarnato, D.; Morandi, E.; Simon, L. M.; Oliviero, S. RNA Framework: An All-in-One Toolkit for the Analysis of RNA Structures and Post-Transcriptional Modifications. *Nucleic Acids Res* **2018**, *46* (16), e97. https://doi.org/10.1093/nar/gky486.

(7)	Darty, K.; Denise, A.; Ponty, Y. VARNA: Interactive Drawing and Editing of the RNA Secondary Structure. *Bioinformatics* **2009**, *25* (15), 1974–1975. https://doi.org/10.1093/bioinformatics/btp250.

(8)	Habibian, M.; Velema, W. A.; Kietrys, A. M.; Onishi, Y.; Kool, E. T. Polyacetate and Polycarbonate RNA: Acylating Reagents and Properties. *Org. Lett.* **2019**, *21* (14), 5413–5416. https://doi.org/10.1021/acs.orglett.9b01526.

(9)	Commichau, F. M.; Gunka, K.; Landmann, J. J.; Stülke, J. Glutamate Metabolism in Bacillus Subtilis: Gene Expression and Enzyme Activities Evolved To Avoid Futile Cycles and To Allow Rapid Responses to Perturbations of the System. *Journal of Bacteriology* **2008**, *190* (10), 3557–3564. https://doi.org/10.1128/jb.00099-08.

(10)	Motika, S. E.; Ulrich, R. J.; Geddes, E. J.; Lee, H. Y.; Lau, G. W.; Hergenrother, P. J. Gram-Negative Antibiotic Active Through Inhibition of an Essential Riboswitch. *Journal of the American Chemical Society* **2020**, *142* (24), 10856–10862. https://doi.org/10.1021/jacs.0c04427.
